# The influence of the gut microbiome on BCG-induced trained immunity

**DOI:** 10.1186/s13059-021-02482-0

**Published:** 2021-09-22

**Authors:** Martin Stražar, Vera P. Mourits, Valerie A. C. M. Koeken, L. Charlotte J. de Bree, Simone J. C. F. M. Moorlag, Leo A. B. Joosten, Reinout van Crevel, Hera Vlamakis, Mihai G. Netea, Ramnik J. Xavier

**Affiliations:** 1 Broad Institute of MIT and Harvard, Cambridge, MA USA; 2 Department of Internal Medicine, Radboud Center for Infectious Diseases (RCI), Radboud University Medical Center, Nijmegen, The Netherlands; 3 Department of Computational Biology for Individualised Infection Medicine, Centre for Individualised Infection Medicine (CiiM) & TWINCORE, Joint Ventures Between the Helmholtz-Centre for Infection Research (HZI) and the Hannover Medical School (MHH), Hannover, Germany; 4 Research Center for Vitamins and Vaccines, Bandim Health Project, Statens Serum Institut, Copenhagen, Denmark; 5 Odense Patient Data Explorative Network, University of Southern Denmark/Odense University Hospital, Odense, Denmark; 6 Department of Medical Genetics, Iuliu Hațieganu University of Medicine and Pharmacy, Cluj-Napoca, Romania; 7 Department for Genomics & Immunoregulation, Life and Medical Sciences Institute (LIMES), University of Bonn, Bonn, Germany; 8 Center for Microbiome Informatics and Therapeutics, Massachusetts Institute of Technology, Cambridge, MA USA; 9 Center for Computational and Integrative Biology, Department of Molecular Biology, Massachusetts General Hospital, Harvard Medical School, Boston, MA USA

**Keywords:** Nonspecific immune responses, Trained immunity, BCG, Microbiome, Phenylalanine metabolism, *Roseburia*, *Eggerthella lenta*

## Abstract

**Background:**

The bacillus Calmette-Guérin (BCG) vaccine protects against tuberculosis and heterologous infections but elicits high inter-individual variation in specific and nonspecific, or trained, immune responses. While the gut microbiome is increasingly recognized as an important modulator of vaccine responses and immunity in general, its potential role in BCG-induced protection is largely unknown.

**Results:**

Stool and blood were collected from 321 healthy adults before BCG vaccination, followed by blood sampling after 2 weeks and 3 months. Metagenomics based on de novo genome assembly reveals 43 immunomodulatory taxa. The nonspecific, trained immune response is detected by altered production of cytokines IL-6, IL-1β, and TNF-α upon ex vivo blood restimulation with *Staphylococcus aureus* and negatively correlates with abundance of *Roseburia*. The specific response, measured by IFN-γ production upon *Mycobacterium tuberculosis* stimulation, is associated positively with *Ruminococcus* and *Eggerthella lenta*. The identified immunomodulatory taxa also have the strongest effects on circulating metabolites, with *Roseburia* affecting phenylalanine metabolism. This is corroborated by abundances of relevant enzymes, suggesting alternate phenylalanine metabolism modules are activated in a *Roseburia* species-dependent manner.

**Conclusions:**

Variability in cytokine production after BCG vaccination is associated with the abundance of microbial genomes, which in turn affect or produce metabolites in circulation. *Roseburia* is found to alter both trained immune responses and phenylalanine metabolism, revealing microbes and microbial products that may alter BCG-induced immunity. Together, our findings contribute to the understanding of specific and trained immune responses after BCG vaccination.

**Supplementary Information:**

The online version contains supplementary material available at 10.1186/s13059-021-02482-0.

## Background

The bacillus Calmette-Guérin (BCG) vaccine consists of the live attenuated microorganism *Mycobacterium bovis* and partially protects against tuberculosis caused by *Mycobacterium tuberculosis* in humans. Epidemiological and experimental data provide evidence that BCG vaccination also protects against heterologous infections [1–3]. This nonspecific protection is at least partially explained by trained immunity, the process in which certain infections and vaccinations result in epigenetic and metabolic rewiring of innate immune cells that increases responsiveness upon subsequent restimulation with unrelated pathogens [4, 5]. In monocytes and natural killer cells, trained immunity induced by BCG vaccination leads to increased production of pro-inflammatory cytokines upon restimulation with nonspecific pathogens, such as *Staphylococcus aureus* and *Candida albicans* [6, 7]. These effects are enabled by modulation of hematopoietic stem and progenitor cells in the bone marrow [8, 9] and last for at least 1 year [10]. However, BCG is not effective in all individuals, and host and environmental factors underlying inter-individual variability of specific and trained immune responses to BCG vaccination are currently incompletely understood [6, 11].

The microbiome plays an essential role in regulating immune responses [12, 13], and numerous mechanisms have been identified through which the microbiome affects immune cell functions, both locally and systemically. For example, microbiome-produced metabolites such as short-chain fatty acids (SCFAs) directly affect myeloid cells [14], while the attachment of segmented filamentous bacteria to the intestinal epithelium improves the function of T helper 17 cells [13, 15]. Moreover, while the microbiome has been proposed to influence the efficacy of vaccines, only a few studies have investigated this [16, 17]. In BCG vaccine responses, two studies observed a role for gut microbiota: mice treated with antibiotics early in life showed impaired antigen-specific IgG responses and T cell generation upon BCG vaccination as well as an increased amount of colony forming units in the lungs upon subsequent *M*. *tuberculosis* infection [18, 19]. Furthermore, a positive association between *Actinobacteria* and BCG vaccine responsiveness in terms of T cell proliferation was observed in samples from 48 infants at 15 weeks of age [20].

Studies investigating the role of the gut microbiome on BCG-induced trained immune responses, however, have not been performed to date. Here, we investigate the effect of the gut microbiota on the induction of specific and nonspecific immune memory responses in a cohort of 321 healthy, BCG-vaccinated individuals. Through associations with the increase in cytokine production after vaccination, we identify potential immunomodulatory species that are further supported and filtered by associations with plasma metabolites. This approach yielded a *Roseburia* genome that showed consistent associations with trained immune responses at two post-vaccination time points and enzyme-mediated effects on phenylalanine metabolites.

## Results

### Microbial profile of study cohort

**Fig. 1:**
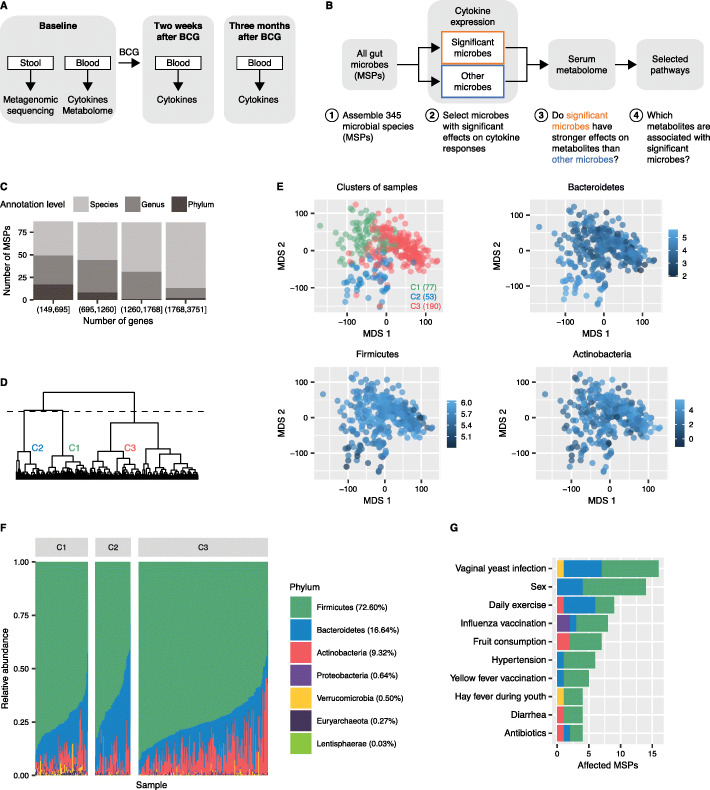
Study design overview and de novo assembly-based analysis of stool microbiome samples. **A** Study design overview. Healthy adult volunteers (*N* = 321) were vaccinated with BCG at baseline, after stool and blood samples were taken for metagenomic, metabolomic, and ex vivo cytokine production analyses. PBMCs isolated from subjects at baseline, after 2 weeks, and after 3 months were stimulated with *S*. *aureus* and *M*. *tuberculosis* ex vivo. **B** Association discovery strategy. Abundance of individual metagenomic species pangenomes (MSPs) was compared to fold changes in cytokine expression after 2 weeks and 3 months. MSPs significant in either modality were subsequently assessed for enrichment of associated circulating metabolites, while metagenomic assembly was searched for enzymes explaining the differential metabolomic potential. **C** Microbiome profiles were quantified as 345 MSP abundances, assembled with MSPminer software. MSPs were matched to known taxa on the phylum (all 345), genus (315), and species (208) levels. Barplot shows annotation levels of MSPs split in quartiles by number of genes. **D** Dendrogram of samples based on Jensen-Shannon distance between relative MSP abundances reveals three clusters. The cutoff point (dotted line) is the maximum mean difference in between-cluster and within-cluster distances (see Figure S1). **E** Multi-dimensional scaling (MDS) plots of samples’ microbial abundances based on Jensen-Shannon distance. Samples are colored by cluster (C1–C3, as in **D**) and cluster sizes are noted in parentheses. The three most abundant phyla (Firmicutes, Bacteroidetes, and Actinobacteria) are highlighted. **F** Per-sample relative abundances of detected phyla. Percentages in parentheses report cohort-wide abundances. **G** Number of MSPs significantly associated with questionnaire variables, stratified by phylum. Color coding as in **F**. The change in relative abundance of each MSP by each individual variable was assessed by Maaslin2 generalized linear model (FDR < 0.25)

To study the influence of the microbiome on BCG-induced trained immunity, we vaccinated 321 healthy individuals of Western European background with BCG [18–75 years old, median age 23 years, 57% female, 83% body mass index (BMI) between 18.5 and 25]. Stool and blood samples were collected at baseline, prior to participants receiving the BCG vaccine. Blood was subsequently collected after 2 weeks and 3 months, as depicted in Fig. 1A. DNA was isolated from the 321 stool samples (“Methods”), amplified, and sequenced using whole genome sequencing. Peripheral blood mononuclear cells (PBMCs) isolated from participants at each time point were subsequently stimulated ex vivo with *S*. *aureus* or *M*. *tuberculosis*; IL-6, IL-1β, and TNF-α were measured after 24 h and IFN-γ was measured after 7 days. Additionally, metabolites were analyzed in plasma from peripheral blood at baseline (Koeken et al. in preparation). Our approach consisted of (1) assembling all microbial species, (2) testing the microbes for modulatory effects on cytokine responses, (3) assessing whether immunomodulatory microbes have stronger effects on circulating metabolites compared to the other microbes, and (4) identifying which metabolites are associated with immunomodulatory microbial species (Fig. 1B).

Reference-based profiling with MetaPhlAn 2.2 detected 202 microbial species, while de novo genome assembly using MSPminer resulted in more than 1.7 million microbial genes. Three hundred and forty-five core metagenomic species pangenomes (MSPs) matched to a phylum, of which 315 (91.3%) and 208 (60.3%) matched to the genus and species level, respectively, with more genes leading to a more precise annotation (Fig. 1C). De novo assembly was subsequently used in all downstream analyses except where explicitly mentioned; besides retrieving all 202 initial species, this method yielded a 1.7-fold increase in the size of the profiled metagenomic space. We hereafter use MSP to refer to a taxonomic unit as a gene set matched to a phylum, genus, or species.

Community-level profiling using core MSP abundances yielded three distinct clusters of samples (Fig. 1D; Additional file 1: Fig. S1A). Average cluster compositions and sample positioning in multi-dimensional scaling plots reflect abundances of the three most common phyla (Fig. 1E). Firmicutes prevailed in cluster 1, with an average relative abundance of 72.6%, followed by Bacteroidetes (16.64%, cluster 2) and Actinobacteria (9.32%, cluster 3) (Fig. 1F). Cluster 1 also showed increased relative abundances of Verrucomicrobia and Euryarchaeota (Additional file 1: Fig. S1B-E). The overall composition reflected that of developed, Western-type societies that tend to be dominated by Firmicutes, while the presence of Bacteroidetes often reflects diet-driven variation [21]. This was corroborated by the species-level profiles being indistinguishable from the independent Dutch 500FG cohort of 473 healthy participants (Additional file 1: Fig. S1F-G) [12].

All the participants in our study were asked to complete a questionnaire that included general health-related questions. Eighteen out of 185 variables associated with the three microbiome clusters (*P* < 0.05, unadjusted Cauchy test), including diet (fruit intake), sex, smoking status, vaccination history, and gastrointestinal status-related variables (Additional file 1: Fig. S2; Additional file 2: Table S1).

Shifts in the relative abundance of individual species correlated with 25 variables (MaAsLin2 generalized linear model, *P*_FDR_ < 0.25), of which 10 correlated with more species (Fig. 1G; Additional file 2: Table S1). In particular, vaginal yeast infection, sex, and daily exercise associated with most microbial species (Fig. 1G). Other species-specific variables that were missed by cluster associations included hypertension, hay fever during youth, and antibiotic use.

### Microbiome species are associated with the capacity to modulate cytokine production after BCG vaccination

**Fig. 2:**
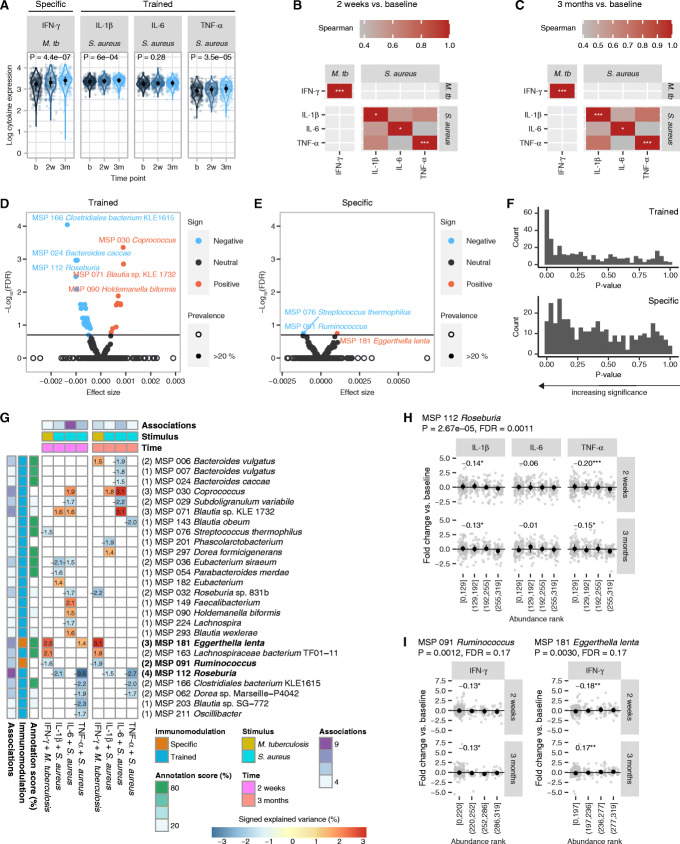
Microbial species abundance changes with differences in trained and stimulus-specific immune responses. **A** Ex vivo cytokine expression upon stimulation with *M*. *tuberculosis* (*M*. *tb*) or *S*. *aureus* at baseline (b) and at 2 weeks (2w) and 3 months (3m) after BCG vaccination. *P*-values were determined by paired Kruskal-Wallis tests. Significant correlations between changes in cytokine expression at 2 weeks (**B**) and 3 months (**C**), measured as fold change against baseline levels (Spearman correlation, *P* < 0.05). Diagonal cells show significance of changes for each individual cytokine versus baseline (*T*-test, *P < 0.05, ***P < 0.001). **D** Significant MSPs associated with trained immune responses subject to FDR < 0.2 and prevalence > 20%. The effect of each MSP was estimated by a linear model of the fold change in TNF-α, IL-1β, and IL-6 responses versus the baseline, adjusted for age and sex (“Methods”). **E** Significant MSPs associated with specific response (IFN-γ stimulated by *M*. *tuberculosis*) and subject to FDR < 0.2 and prevalence > 20%, using a model of the same form as in **D**. **F** Distribution of unadjusted *P*-values for each MSP in the trained and specific response models. Skew from uniform distribution suggests more pronounced effects of MSPs on cytokine expression fold change. **G** Individual effects on cytokine fold changes by each MSP and time point, quantified as explained variance multiplied by effect sign. Number of associations per MSP and confidence in species-level annotation are reported on the left. **H** MSP 112 (*Roseburia*) abundance decreases two of the three trained immunity phenotypes in both subsequent time points. Relative abundance of the MSP was converted to ranks, and ranks were sorted into bins by equal-frequency binning. *P*-values in titles refer to the trained immunity linear model. Coefficients in panels are Spearman correlations (*P < 0.1, ***P < 0.001). **I** MSP 091 (*Ruminococcus*) and MSP 181 (*Eggerthella lenta*) ranked abundance versus specific response phenotype. Relative abundance of the MSP was converted to ranks, and ranks were sorted into bins by equal-frequency binning. *P*-values in titles refer to the specific response linear model. Coefficients in panels are Spearman correlations (*P < 0.1, **P < 0.01)

Cytokine production in the supernatant of stimulated PBMCs was measured to assess the trained immune response (IL-6, IL-1β, TNF-α after 24-h stimulation) and the specific immune response (IFN-γ after 7-day stimulation). IL-1β and TNF-α production upon *S*. *aureus* stimulation and IFN-γ production upon *M*. *tuberculosis* stimulation gradually increased 2 weeks and 3 months after BCG vaccination compared to baseline (Fig. 2A; Additional file 1: Fig. S3A). Trained immunity (indicated by increases in TNF-α, IL-1β, IL-6 production) as well as specific (*M*. *tuberculosis*-induced IFN-γ) and heterologous lymphocyte-derived (*S*. *aureus*-induced IFN-γ) cytokine responses correlated with each other (Fig. 2B, C). The microbiome clusters did not show a significant divergence in any of the cytokine responses measured upon ex vivo stimulation of PBMCs (maximum *P* = 0.16 for IFN-γ with *S*. *aureus*, Additional file 1: Fig. S3B), prompting the search for a potential individual taxon or functional immunomodulatory factors.

The associations between microbes and both immune system modalities were assessed with two linear models, adjusted for age and sex, testing for effects on trained immunity and specific T cell responses (“Methods”). Trained immunity was associated with 40 significant species (prevalence > 20%, FDR < 0.2, Fig. 2D; Additional file 3: Table S2), while specific immunity was associated with three species: *Ruminococcus* (MSP 091), *Eggerthella lenta* (MSP 181), and *Streptococcus thermophilus* (MSP 076) (Fig. 2E; Additional file 3: Table S2). At FDR < 0.1, 27 species were associated with trained immunity. Because the model for the trained immunity response combined more biological markers (three cytokines) than that for the specific response (one cytokine), it retrieved more species at the same significance threshold. Nevertheless, both modalities elicited a non-uniform distribution of p-values (Fig. 2F), supporting an influence of the microbiome on BCG vaccine responses.

We next examined the correlation between species relative abundance and individual cytokine production at both time points after vaccination (Fig. 2G). A *Roseburia* genome (MSP 112), only identifiable on the genus level, was negatively correlated with the increase in IL-1ß and TNF-α production at both time points (*P* = 2.67e−05, FDR = 0.0011, Fig. 2H). In the specific response model, *Ruminococcus* (MSP 091, *P* = 0.0012, FDR = 0.17) and *E*. *lenta* (MSP 181, *P* = 0.0030, FDR = 0.17) showed consistent negative and positive effects, respectively, on IFN-γ at both time points (Fig. 2I). The specific response model revealed the most associations between a single cytokine and microbial species, although for all the cytokine–stimuli combinations, the number of unadjusted associations ranged between 10 and 23 microbes (Additional file 4: Table S3).

### Immunomodulatory microbes strongly influence circulating metabolites

Detectable changes in cytokine production yielded potential immunomodulatory species, whose exact numbers varied with selected statistical thresholds. To corroborate cytokine associations and narrow down the potential causal mechanisms, we compared the effects of immunomodulatory microbes on the plasma metabolome, measured as previously described [59]. A total of 1607 mass/charge (m/z) peaks were matched to known compound identities and clustered into 20 groups, enriched for eight major molecular classes (Additional file 1: Fig. S4A-C, “Methods”).

**Fig. 3:**
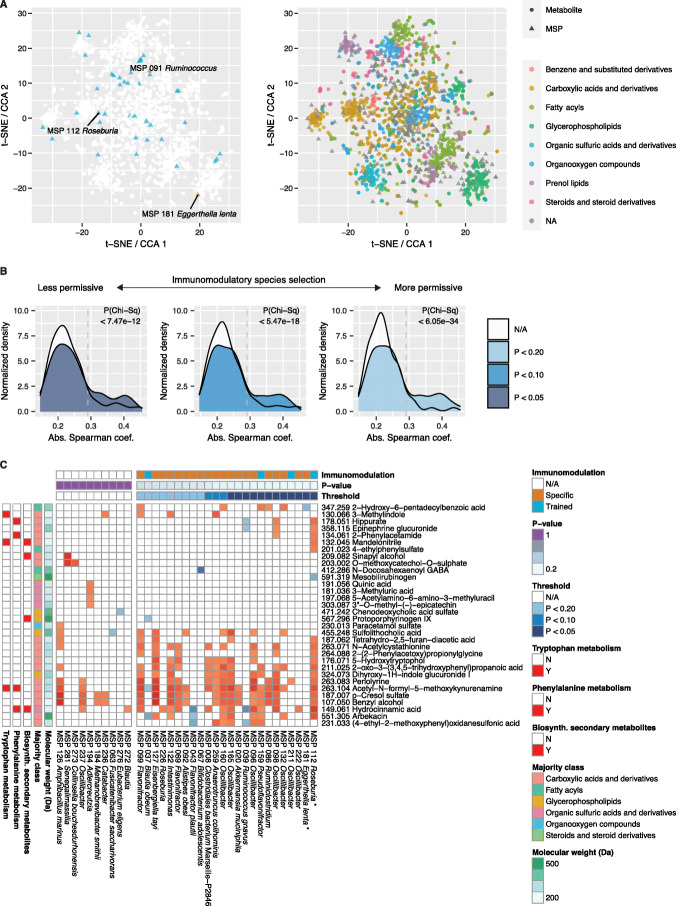
Immunomodulatory species strongly influence the serum metabolome. **A** Canonical correlation analysis (CCA) between abundances of the 345 MSPs and 1607 annotated metabolites across all 321 samples. Twenty-five canonical components with significant increase in explained variance were projected in two dimensions using t-SNE. Positions of three immunomodulatory MSPs (left) and metabolite classes (right) are highlighted. Joint deviation of points from the origin (coordinate 0, 0) reflects increased covariance and grouping between metabolites and MSPs. **B** MSPs showing effects on cytokine responses have larger effects on metabolite intensities. Distribution of maximum absolute Spearman correlations per MSP across all metabolites. MSPs present in at least 20% of the samples and significant subject to *P* < 0.05 (left), < 0.1 (center), and < 0.2 (right) in any of the two log-linear models are shown in blue and the remaining non-significant MSPs are in white. Dotted vertical line shows threshold for significantly correlated species and metabolites (*P*_Bonferroni_ < 0.05). **C** All 32 significantly associated metabolites by 34 MSPs. Colored heat map cells show Spearman correlations with *P*_Bonferroni_ < 0.05. Columns (MSPs) are sorted by minimum *P*-value from any of the two log-linear models. Asterisks indicate MSPs associated in cytokine responses shown in Fig. 2

Canonical correlation analysis confirmed a strong microbiome effect on compound intensities, as 15 of the top 25 canonical components (CCs) showed significant explained variance (Additional file 1: Fig. S4D-E). A t-SNE projection of the top 25 CCs showed strong grouping by molecular class, with evident co-occurrence of microbes in clusters harboring carboxylic acids and derivatives, glycerophospholipids and organic sulfuric acids (Fig. 3A). The three immunomodulatory species *Roseburia* (MSP 112), *Ruminococcus* (MSP 091), and *E*. *lenta* (MSP 181) strongly influenced the composition of the metabolite space, as seen by their polarized placement in the joint projection (Fig. 3A).

Next, we investigated whether immunomodulatory microbes have stronger effects on individual metabolites compared to the other microbes. All pairwise Spearman correlations yielded 198 significant associations (*P*_Bonferroni_ < 0.05) between 34 MSPs and 32 metabolite intensities (Additional file 5: Table S4). While most associations were positive (183/198), the resulting increase in metabolite intensities might result both from microbe-mediated anabolism or compound breakdown. To test whether species with large effects in either of the two linear models are more likely to elicit significant effects on the metabolome, we used three different thresholds that selected at least 40 species and computed the largest absolute effect on any one metabolite. All three settings yielded an enrichment of significant effects among immunomodulatory microbial species (maximum *P* < 10^−11^, Chi-square test, Fig. 3B). This confirmed that microbial species with effects on cytokine production also have strong effects on circulating metabolites.

Of the 198 significant metabolite associations, 18 were associated with the presence of *Roseburia* (MSP 112), the most of any MSP, and one with *E*. *lenta* (MSP 181) (Fig. 3C). Due to uncertainty in annotation and pathway-mediated dependence on metabolite abundance, we attempted to identify general mechanisms by grouping the metabolites using KEGG pathways. Phenylalanine metabolism was influenced most strongly by *Roseburia* (MSP 112), with four metabolite associations in this pathway. *Roseburia* (MSP 112) additionally influenced three metabolites each in tryptophan metabolism and biosynthesis of secondary metabolites (Fig. 3C).

Untargeted plasma metabolomics detected two SCFAs, acetate and propionate, which are microbial products of dietary fiber metabolism and candidate mediators of host–microbiome signaling [14]. Both molecules showed significant positive associations with trained immunity but not with the specific response (Additional file 1: Fig. S5). While immunomodulatory microbes were not significantly correlated with either acetate or propionate, *E*. *lenta* had the highest positive correlation with propionate.

Butyrate, an SCFA previously shown to decrease IL-6 production [22], was not detected in circulation, possibly due to its absorption in the gut [23]. Nevertheless, butyrate kinase, which catalyzes the last step of butyrate biosynthesis [24], was detected in a single *Roseburia* (MSP 209) and five *Coprococcus* species, where *Coprococcus* (MSP 030) had the strongest positive association with trained immunity, in particular an increase in IL-6, after vaccination (Additional file 1: Fig. S6A-C). Conversely, *Coprococcus comes* (MSP 213) correlated with a decreased trained immunity response, manifested in reduced TNF-α expression after 3 months (Additional file 1: Fig. S6D). The absence of butyrate kinase in immunomodulatory *Roseburia* (MSP 112) may stem from gaps in assembled genomes or allow for metabolic modalities other than SCFAs in mediating BCG-induced immunity.

Combined evidence from the (i) modeled effects of microbes on cytokine production, (ii) associations between species relative abundances and the production of two trained immunity-related cytokines at both time points after vaccination, and (iii) effects of the immunomodulatory microbiome species on the plasma metabolome yielded the prediction that *Roseburia*-mediated phenylalanine metabolism influences trained immunity.

### *Roseburia* encodes phenylalanine pathway enzymes that are prevalent and abundant

**Fig. 4:**
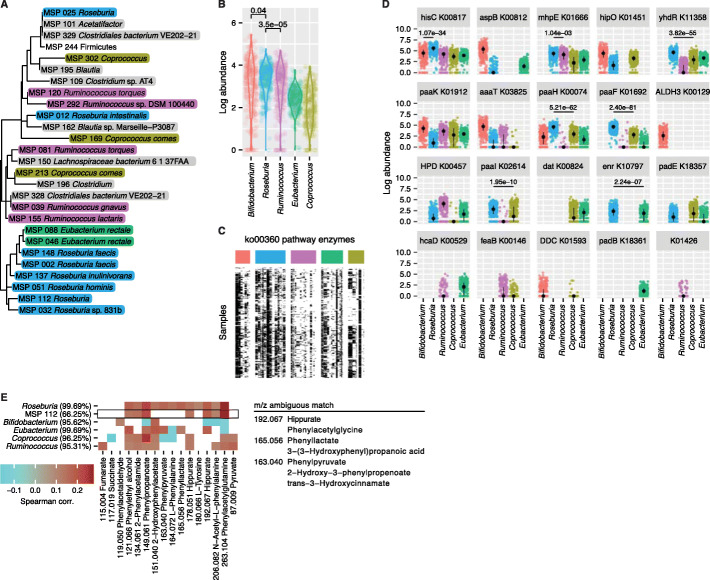
Phenylalanine pathway enzymes encoded by *Roseburia* are prevalent and abundant. **A** A subtree of the phylogenetic relationships among detected MSPs, containing the majority of MSPs mapped to the *Roseburia* genus (PhyloPhlAn 3.0). **B** Total abundance of each genus in the cohort. **C** Assembled genes encoding 20 of the 77 enzymes in the phenylalanine metabolism pathway (KEGG ortholog ko00360) across samples, sorted by MSPs in each genus. Heatmap shows samples where a gene is detected. **D** Total abundance of individual genes in the phenylalanine metabolism pathway. For each KEGG ortholog group, statistics of all assembled homologs were aggregated. Each enzyme encoded by *Roseburia* was tested for increased abundance (read per million mapped reads, RPKM) compared to the other four genera in the panel. The minimal *P*-value from up to four comparisons is shown for the cases where *Roseburia* has the highest abundance. Black points show the median, and the error bars mark the 25th and 75th percentiles. **E** Spearman correlations of MSPs in the panel with 16 detected metabolite features (mass-to-charge, m/z, peaks in mass spectra) mapping to the phenylalanine metabolism pathway. Only the top matching compound name is shown, while ambiguous matches are shown on the right. For each genus, the strongest effect associated with any relevant MSP was chosen and marked as significant subject to *P* < 0.05. The effects associated with MSP 112 (*Roseburia*) are highlighted in the black box

The effects on phenylalanine metabolites prompted us to investigate the *Roseburia* genomes and confirm the presence of relevant enzymes. We compared the differential enzymatic and metabolic potential of *Roseburia* to phylogenetically and biochemically related taxa by a deeper profiling of the stool metagenomes. All 345 identified MSPs were grouped by phylogenetic divergence using core genes (PhyloPhlAn 3.0, Additional file 1: Fig. S7), revealing a clade harboring most *Roseburia* species (Fig. 4A). The phylogenetically closest Firmicutes included five *Ruminococcus*, three *Coprococcus* and two *Eubacterium rectale* MSPs. While *E*. *rectale* is related to *Roseburia* in terms of butyrate production, we also included a biochemically distinct but abundant Actinobacteria *Bifidobacterium* (MSP 111). The assembled panel consisted of 59 MSPs derived from 85,208 assembled genes of *Bifidobacterium* (7 MSPs), *Roseburia* (13 MSPs), *Ruminococcus* (17 MSPs), *Eubacterium* (15 MSPs), and *Coprococcus* (7 MSPs), with each genus having more than 95% prevalence in the cohort (Fig. 4B).

A total of 330 genes mapped to 20 out of 77 enzymes in the phenylalanine pathway (KEGG ko00360). *Roseburia* contributed the most genes (92, 45.1% average prevalence; Fig. 4C; Additional file 6: Table S5), whereas other genera in the panel contributed fewer [*Ruminococcus* (77, 19.2%), *Eubacterium* (66, 36.3%), *Coprococcus* (50, 35.5%) and *Bifidobacterium* (45, 46.1%)]. *Roseburia* also encoded the most phenylalanine enzymes (13 out of 20). Of these, *hisC*, *mhpE*, *yhDR*, *paaH*, *paaF*, *paaI*, and *enr* were significantly more abundant in *Roseburia* than in all of the other four genera (Fig. 4D).

A total of 15 m/z peaks matched to compounds from the phenylalanine pathway (KEGG ko00360), potentially spanning up to 19 compounds due to ambiguous matches. Species from the *Roseburia* genus significantly correlated with 11 of the 15 compounds in the pathway, of which MSP 112 contributed to six of these compounds (Spearman correlation, *P* < 0.05, Fig. 4E). The genera *Ruminococcus*, *Coprococcus*, *Bifidobacterium*, and *Eubacterium* each showed fewer associations (Fig. 4E). Taken together, *Roseburia*’*s* increased influence on the phenylalanine metabolism pathway compared to closely related species is confirmed through increased enzymatic potential and metabolite intensity.

### Comparison of *Roseburia* genomes reveals alternate routes in phenylalanine metabolism

**Fig. 5:**
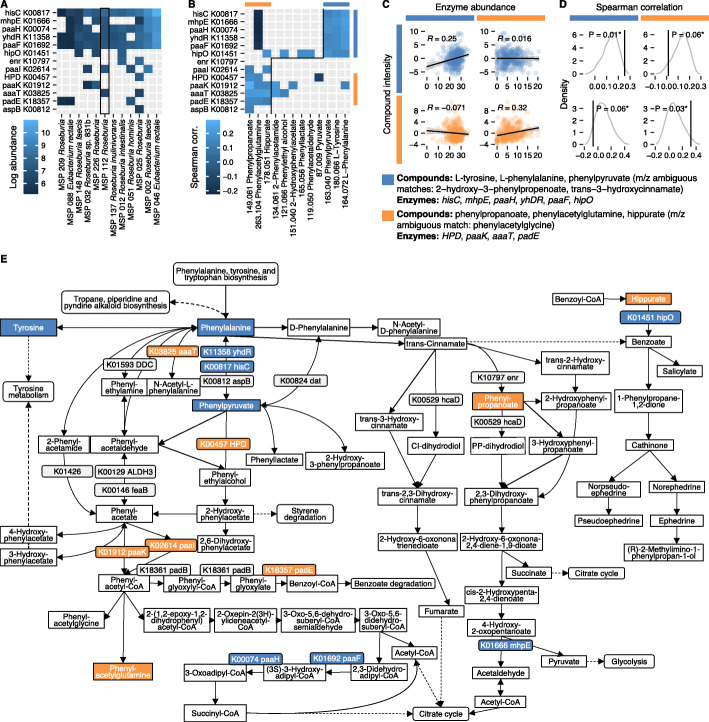
Integrative analysis of *Roseburia* effects on the phenylalanine metabolism pathway (KEGG ko00360). **A** Total abundances of enzymes encoded by MSPs in the *Roseburia* genus, with MSP 112 highlighted in the black box. **B** Observed enzymes and compounds in the phenylalanine pathways. Compounds and enzymes in blue and orange groups are colored accordingly. Significant Spearman correlations between enzymes encoded by *Roseburia* and phenylalanine metabolism compound intensities. Only the top matching compound name is shown. Compound enzyme groups are indicated by orange and blue bars. The black line emphasizes the separation between the two groups. **C** Correlation between total abundances of enzymes and average intensities of compounds grouped by blue and orange bars in **B**. **D** Null distribution of correlations between selected compounds and random groups of six (blue group) or five genes (orange group) from the *Roseburia* genomes. Observed Spearman correlations (as in **C**) are marked with a black vertical line and the null distribution is shown in gray. **E** Phenylalanine metabolism pathway (KEGG ko00360). Detected compounds and enzymes are colored in blue and orange

Finally, we more closely investigated the 13 phenylalanine metabolism enzymes found within the *Roseburia* genus and their differential effects on circulating metabolites. Five of the enzymes (*hisC*, *mhpE*, *paaH*, *yhDR*, and *paaF*) were common to over half of the MSPs, while the remaining eight were more species-specific (Fig. 5A). The co-occurrence of *aaaT* (L-phenylalanine N-acetyltransferase) and *paaI* (acyl-CoA thioesterase) was specific to the immunomodulatory MSP 112, as they were not found together in any other *Roseburia* MSP. These enzymes act in the path from phenylalanine to N-acetyl-L-phenylalanine and phenylacetylglutamine, whose plasma concentrations were associated with increased *Roseburia* (MSP 112) abundance (Fig. 4E).

Correlations between individual enzyme abundances and compound intensities revealed two distinct pathway modalities. First, an increase in L-tyrosine, L-phenylalanine, and phenylpyruvate (m/z ambiguous matches: 2−hydroxy−3−phenylpropenoate, trans−3−hydroxycinnamate) was accompanied by the five commonly shared enzymes (*hisC*, *mhpE*, *paaH*, *yhDR*, and *paaF*) as well as *hipO* (grouped by blue bars in Fig. 5B). Second, *HPD*, *paaK*, *aaaT*, and *padE* were associated with an increase in phenylpropanoate, hippurate (m/z ambiguous match: phenylacetylglycine), and phenylacetylglutamine (grouped by orange bars in Fig. 5B). The latter compounds also strongly decreased with the presence of the five shared enzymes, suggesting an opposing mechanism between the two groups of compounds and enzymes.

We assessed the significance of the observed correlations to verify that the differences in compound intensities in each group were indeed more likely due to the associated enzymes than any other genes. The Spearman correlation between sums of enzyme abundances and compound intensities within each group revealed even stronger effects than any individual compound–enzyme association (Fig. 5C, top-left and bottom-right panels). Conversely, correlations between groups showed weak to negative effects (Fig. 5C, top-right and bottom-left panels). Finally, the observed correlations were compared to a null distribution of correlations from 10,000 random draws of six (blue group) or four (orange group) genes from all 22,832 *Roseburia* genes. Indeed, all four observed effects were extreme when compared to random sets of genes (Fig. 5D), confirming that the opposing compound–enzyme associations are unlikely to be due to other *Roseburia* genes. The complete sets of detected compounds and enzymes are shown in Fig. 5E, with group separation indicated. These results reveal two mechanisms, each characterized by a mutually exclusive set of enzymes and compounds, outlining how species-level differences in *Roseburia* may affect phenylalanine metabolites in circulation.

Additional evidence for the capability of *Roseburia* to alter compounds in the phenylalanine metabolism pathway is provided by a study that performed targeted metabolomics using reference standards on supernatants of individual microbial species grown in defined media [25], including *Roseburia inulinivorans*, a close relative of *Roseburia* (MSP 112) with species-level annotation (Fig. 4A). A change of at least twofold in *R*. *inulinivorans* supernatant was observed for two compounds: an increase in 2-hydroxyacetate, which is encoded downstream of *yhDR* and *hisC*, and a decrease in succinate, which is encoded upstream of *mphE* (Additional file 1: Fig. S8, Fig. 5D). Although these compounds were not detected using our plasma metabolomics methods, the direction of changes is aligned with the relevant genes and supports an active role of *Roseburia* in phenylalanine metabolism pathways.

## Discussion

Growing evidence suggests that the gut microbiome mediates effects on vaccine efficacy through microbial ligands, circulating metabolites or both. Here, we identified microbial species associated with BCG-induced specific (adaptive immune memory) and heterologous (trained immunity) responses. De novo assembly methods and associations with circulating metabolites and cytokines at multiple time points enabled us to obtain statistically robust microbial signals that would otherwise be missed by reference-based methods.

We uncovered a total of 43 immunomodulatory microbes, including two species that showed consistent effects through metagenomic and metabolomic analyses: *Roseburia* (MSP 112) negatively associated with cytokine responses upon BCG-induced nonspecific immunity, while *Eggerthella lenta* positively associated with BCG-induced specific T cell-mediated memory responses. To date, the only other study investigating the BCG vaccination and human gut microbiota was performed with a cohort of 48 infants from Bangladesh [20]. Positive associations were observed between Actinobacteria (including *Bifidobacterium*) and immune responses, as measured by T cell proliferation and delayed-type hypersensitivity skin test, 15 weeks after BCG vaccination at birth. Although it is difficult to extrapolate these results to our cohort, we also observed that a genus of Actinobacteria (*Eggerthella*) was associated with specific T cell immune responses upon BCG vaccination.

Microbial products and cell wall components such as low-dose lipopolysaccharides (LPS) are known to modulate immune response and tolerance through engagement of host pattern recognition receptors [26]. Trained immunity responses in particular were shown to be dependent on engagement of the NOD2 receptor by muramyl dipeptide (MDP), the active component of mycobacterial peptidoglycans [27], which positively correlated with changes in IL-6, IL-1β, and TNF-α upon *S*. *aureus* stimulation 3 months after BCG vaccination [28]. As MDP can originate from the microbiome [29], it is unsurprising to detect numerous microbial associations with BCG-induced trained immunity (Fig. 2D, E).

*Roseburia* species produce SCFAs such as butyrate, which are known to possess immunomodulatory properties [30]. Interestingly, butyrate supplementation reduces induction of trained immunity [31], which could be speculated to be an additional mechanism through which *Roseburia* modulates trained immunity. Associations between *Roseburia* and atherosclerosis [32] — a process in which trained immunity also plays an important role [33] — as well as between *Eggerthella* and rheumatoid arthritis [34], multiple sclerosis [35], and inflammatory bowel disease [36] further suggest immunomodulatory roles for these species. Moreover, *E*. *lenta* is often associated with serious gastrointestinal infections [37] and has been shown to increase intestinal T helper 17 cell numbers and worsen murine models of colitis.

Upon analyzing microbiome composition in conjunction with health-related variables from a questionnaire, we identified correlations between variations in the intestinal microbiome and adult vaccinations against influenza, yellow fever, and HPV. However, we are unable to determine whether these vaccinations altered microbiome composition or whether different lifestyles associated with additional vaccinations contributed to differences in the microbiome. In line with this, changes in the microbiome associated with yellow fever and influenza vaccination also coincided with participants traveling outside Europe. Variables including history of vaginal fungal infections, sex, diet, diarrhea, antibiotic use, and daily exercise also correlated with the number of changes in species relative abundance. The latter five factors are known to impact the gut microbiome [38–42]. Interestingly, hay fever during youth was associated with *Verrucomicrobia*, which has been observed at higher abundance in infants with allergy [43]. While gut microbiome profiles correlated with a number of health-related variables, overall metabolic health (e.g., BMI) did not correlate with cytokine responses upon stimulation, likely since our cohort is composed of healthy young adults (median age 23 and BMI = 25 at the 85th percentile).

The immunomodulatory microbes we identified, in particular *Roseburia* (MSP 112), had a stronger impact on circulating metabolites than the other microorganisms, suggesting a mechanism through which immune responses are influenced. Of the metabolites associated with metagenomic species, several were already known to be produced or modified by the gut microbiome, including acetyl-N-formyl-5-methoxykynurenamine (AFMK) [44], p-cresol sulfate [45], 5-hydroxytryptophol [46], hydrocinnamic acid [47], 4-ethylphenyl sulfate [48], hippurate [49], mandelonitrile [50], and 3-methylindole [51].

Most of the metabolites corresponding with microbiome changes were related to phenylalanine, an essential amino acid involved in many processes and that is also converted into tyrosine. Tyrosine comprises catecholamines and is involved in fumarate production, both of which are known to induce trained immunity responses [52]. Phenylalanine to tyrosine synthesis is facilitated by cofactor BH_4_, whose availability is influenced by pro- and anti-inflammatory stimuli in human macrophages. In general, increased serum phenylalanine concentrations are found during immune activation in humans, most likely due to the reduced converting rate. In phenylketonuria, a human congenital disorder in which a mutation in phenylalanine hydroxylase leads to high phenylalanine concentrations, a deficit of tyrosine and catecholamines is observed [53]. Microbial derivatives of phenylalanine and tyrosine, including microbiota-dependent metabolites p-cresol sulfate and phenylacetylglutamine [54], are known to contribute to chronic kidney disease pathogenesis and disease progression by inducing renal damage and inflammation [55, 56]. Furthermore, these metabolites have been associated with overall mortality and cardiovascular disease in individuals with kidney disease [54], and phenylacetylglutamine is a risk factor for major adverse cardiovascular events [57].

The *Roseburia* genus was associated with 11 out of 15 compounds in the phenylalanine pathway, with MSP 112 having the strongest effect. Moreover, the *Roseburia* genus encodes enzymes in the phenylalanine metabolism pathway itself. Related Firmicutes *Ruminococcus*, *Eubacterium* and *Coprococcus*, as well as *Bifidobacterium* displayed decreased enzyme presence compared to *Roseburia*. The enzymes detected in MSP 112 and absent in the majority of other *Roseburia* genomes were L-phenylalanine N-acetyltransferase (*aaaT*), acyl-CoA thioesterase (*paaI*), and 4-hydroxy 2-oxovalerate aldolase (*mhpE*), which are found at different steps in the phenylalanine pathway. Notably, *mhpE* is involved in the production of pyruvate [58], and although pyruvate was not associated with *Roseburia* itself, it is used during trained immunity to increase metabolic activity [21].

Limited sensitivity and coverage of current metagenomic methods do not exclude the existence of additional, undetected immunomodulatory microbes. Nevertheless, associations with cytokine expression, circulating metabolites, and differential presence of phenylalanine metabolism enzymes increase the confidence that the presence of *Roseburia* in the gut microbiome dampens trained immune responses after BCG vaccination. Further investigation of the role of phenylalanine metabolism in these responses is warranted in future studies, as a possible causal role between *Roseburia* species and phenylalanine metabolism is supported by recently published in vitro experiments and should continue to be investigated in similar controlled settings. Furthermore, since individuals included in this study were all from Western European ancestry, of adult age, and living in similar environments, these findings should be validated in additional cohorts of different age groups and of different genetic backgrounds. This study nevertheless provides a detailed insight into associations between assembled microbial genomes, plasma metabolites and subject-specific immune responses.

## Conclusions

The data presented here details for the first time microbiome-associated effects on BCG vaccine responsiveness in a large human cohort. The pathways and microbial species that mediate BCG-induced trained and specific immune responses contribute to the understanding of subject-specific immunity and may inform personalized strategies to enhance vaccine efficacy.

## Methods

### Experimental model and subject details

In the 300BCG study, 321 healthy Dutch individuals were included from April 2017 until June 2018. Individuals were not included if they did not have a Western European ancestry, used systemic medication other than oral contraceptives and acetaminophen, used antibiotics 3 months before inclusion, received previous BCG vaccination, had a history of tuberculosis, any febrile illness 4 weeks before participation, vaccination 3 months before participation, or a medical history of immunodeficiency. Individuals were excluded for not meeting inclusion criteria (*n* = 2), medication use (*n* = 4), different time of vaccination (*n* = 18), or a missing stool sample (*n* = 1). At the hospital, written informed consent was obtained, stool and blood samples were collected, and participants were vaccinated with a standard dose of 0.1 mL BCG Bulgaria (InterVax) intradermally in the left upper arm. At 2 weeks and 3 months after vaccination, blood was again collected and a questionnaire was completed. This study was approved by the Arnhem-Nijmegen Medical Ethical Committee (NL58553.091.16) and performed in accordance with the Declaration of Helsinki.

### Method details

#### PBMC isolation and stimulation

Ficoll-Paque (GE Healthcare, UK) density gradient separation was used to isolate peripheral blood mononuclear cells (PBMCs) from EDTA whole blood. After washing twice with phosphate buffered saline (PBS), cells were counted with the Sysmex hematology analyzer (XN-450) and resuspended in culture medium consisting of Dutch modified RPMI 1640 (Invitrogen, USA) supplemented with 50 μg/mL gentamicin, 2 mM Glutamax (Gibco Life Technologies), and 1 mM pyruvate (Gibco Life Technologies). Five hundred thousand PBMCs per well were added to a round bottom 96-well plate (Greiner) and stimulated with culture medium (control), heat-killed *Mycobacterium tuberculosis* HR37v (5 μg/mL), or heat-killed *Staphylococcus aureus* (10^6^ CFU/mL, clinical isolate), and incubated at 37 °C, 5% CO_2_, for 24 h or 7 days. Medium of 7 days stimulation was supplemented with 10% human pooled serum. After 24 h and 7 days, supernatants were collected and stored at −20 °C until further analysis.

#### Cytokine measurements

IL-1β, IL-6, and TNF-α (R&D Systems) concentrations were measured in 24-h supernatants with ELISA, according to the manufacturer’s protocol. IFN-γ was measured in 7-day supernatants with Luminex (ProcartaPlex, Thermo Fisher), according to the manufacturer’s protocol. The cytokine concentrations were measured in pg/mL, adjusted for plate variation using linear model residuals, and log-transformed.

#### Untargeted plasma metabolomics

Plasma was obtained from participants by centrifugation of EDTA blood and was immediately stored at −80°C until further analysis. Flow injection-time-of-flight mass (flow injection TOF-M) spectrometry was performed by General Metabolomics (Boston, USA), as previously described [59]. A list of putative metabolites was annotated with a series of analysis strategies including deisotoping, decluttering, adduct detection, and library matching in KEGG, HMDB and CHEBI databases, resulting in 1,607 annotated features. All metabolomic measurements were performed in duplicate, and the average value was calculated for each sample per feature.

#### Stool samples collection and sequencing

Stool samples were collected at home the day before or on the day of blood collection, time was recorded, and samples were stored in the refrigerator at home. At the hospital visit before BCG vaccination, samples were immediately aliquoted and stored at −80 °C.

Nucleic acids were extracted the AllPrep 96 PowerFecal DNA/RNA kit from QIAGEN (custom product # 1114341). This method pairs bead-beating on a Tissuelyser II (QIAGEN) with a 96-well AllPrep protocol and is available through QIAGEN. Bead-beating is performed twice at 20 Hz for 5 minutes each round with a rotation of the plate in between rounds. Purified DNA was stored at –20 °C. Metagenomic sequencing libraries were prepared from 2 ng of input DNA using the Nextera XT DNA Library Preparation kit (Illumina) according to the manufacturer’s recommended protocol. Prior to sequencing, libraries were pooled by collecting equal volumes of each library. Insert sizes and concentrations for each pooled library were determined using an Agilent Bioanalyzer DNA 1000 kit (Agilent Technologies) prior to sequencing on an Illumina NovaSeq 6000 with 151 bp paired-end reads to yield ~ 10 million paired-end reads per sample. Data was analyzed using the Broad Picard Pipeline which includes de-multiplexing and data aggregation (https://broadinstitute.github.io/picard).

### Quantification and statistical analysis

#### Metagenomics data processing

The quality control for sequencing data was conducted using Trim Galore! to detect and remove sequencing adapters (minimum overlap of 5 bp) and kneadData to remove human DNA contamination and trim low-quality sequences (HEADCROP:15 SLIDINGWINDOW:1:20), retaining reads that were at least 50 bp. Metagenomic reads from all cohorts were assembled individually for each sample into contigs using MegaHIT [60], followed by an open reading frame prediction with Prodigal [61] and retaining only full length genes (containing both start and stop codon).

A non-redundant gene catalog was constructed by clustering predicted genes based on sequence similarity at 95% identity and 90% coverage of the shorter sequence using CD-HIT [62]. Reads were mapped to the gene catalog with BWA [63], filtered to include strong mappings with at least 95% sequence identity over the length of the read, counted (count matrix) and normalized to transcript-per-million (TPM matrix). Count matrix served as an input for binning genes into metagenomic species pan genomes (core and accessory genes) using MSPminer with default settings [64].

The abundance of an MSP was quantified as median gene TPM. The genes forming each MSP were quantified as reads per kilobase (RPK).

We annotated the gene catalog at species, genus, and phylum levels with NCBI RefSeq (version May 2018) as described previously [65]. The KEGG Orthogroups were assigned to each gene in the catalog using eggNOG-Mapper [66].

The PhyloPhlAn 3.0 with default settings [67] was used to organize the phylogenetic tree of MSPs based on similarity in core genes.

For comparison with the 500FG cohort, we used the sample pipeline as described in the associated publication [12]. After the kneadData quality control step, marker-gene based taxonomic annotation tool MetaPhlAn [68] yielded relative abundances of 202 species, of which 199 were shared between the two cohorts.

#### Clustering of metagenomic species

The abundances of MSPs were normalized to relative abundance in each sample. The clusters were determined based on the Jensen-Shannon divergence and a consensus hierarchical clustering with pvclust using 100 bootstrap runs. The number of clusters was determined to maximize the difference of average between- and within-cluster divergences.

#### Variable associations with metagenomic species

We tested variables known for at least 200 samples and had a minimum 40 samples per categorical variable level. Maaslin2 generalized linear model was used to model differential abundance of each MSP and the recommended threshold of *P*_FDR_ < 0.25 was applied.

#### Significance of cytokine expression changes after vaccination

Significance of increased log cytokine expression over three time points was assessed using paired Kruskal-Wallis test. The *T*-test was used to determine significance of fold changes from the baseline time point for 2 weeks and 3 months. Spearman correlation was used to test for significant correlations between cytokine levels.

#### Associations between metagenomic species and changes in cytokine expressions

To find potential immunomodulatory species affecting the magnitude in the immune response after BCG vaccination, we combined the data on cytokine expression over three time points with stool samples at baseline and subjects’ age and sex. We included 303 samples vaccinated in the morning and at least two cytokine measurements (*i* = 1...303) and 163 metagenomic species (MSPs) with at least 20% prevalence (*m* = 1...202). Cytokine expressions (c) were quantified as fold change against the baseline for measurements after 2 weeks and 3 months (*t* = 2 weeks, 3 months). To equalize the variance in cytokine expressions, the fold changes were standardized for each cytokine and time point using the z-score transform.

Trained and specific responses were assessed with two separate models. The cytokines TNF-α, IL-1β and IL-6 stimulated with *S*. *aureus* were used to quantify the trained immune response, while IFN-γ stimulated with *M*. *tuberculosis* was used to detect the specific immune response. Excluding missing values, the trained immunity model comprised 1685 observations, while the specific response model comprised 535 observations. For both response types the *z*-scored fold changes *z*_c,t,i_ were fit using the linear model

$$ {\mathrm{z}}_{\mathrm{c},\mathrm{t},\mathrm{i}}={\upalpha}_0+{\upalpha}_{\mathrm{a}}{\mathrm{a}\mathrm{ge}}_{\mathrm{i}}+{\upalpha}_{\mathrm{s}}{\mathrm{s}\mathrm{ex}}_{\mathrm{i}}+{\upalpha}_{\mathrm{m}}{\mathrm{m}\mathrm{sp}}_{\mathrm{m},\mathrm{i}}+{\upvarepsilon}_{\mathrm{c},\mathrm{t},\mathrm{i}}, $$

with the following parameters: *ε*_c,t,i_ is the unexplained variance, *α*_0_ the intercept, and *α*_a_, *α*_s_, *α*_m_ coefficients for age, sex, and MSP abundance, respectively. The subject-specific variables are age_i_ (continuous), sex_i_ (discrete), and msp_m,i_ the relative of the MSP *m*, converted to ranks (where no abundance amounts to rank 0 and maximum abundance to rank 302). Note that there is no separate longitudinal or cytokine coefficient, as we sought species that associate with responses in all cytokines at both time points, effectively treating the measurements as replicates in order to decrease model complexity and increase statistical power. Despite the observations not being entirely independent (each subject can be repeated in multiple time points and cytokines), we chose to treat them as such rather than average across cytokines and timepoints, treating them as separate experiments that bear additional information.

To account for co-abundance effects, each of the two models was fit once for each MSP, using the R function lm. The MSPs were deemed immunomodulatory if *α*_m_ had a significant contribution after multiple testing adjustment (*P*_FDR_ < 0.2, *T*-test). The explained variance in cytokine expression fold change was computed for each MSP, using the model based on age and sex alone:

$$ {\mathrm{z}}_{\mathrm{c},\mathrm{t},\mathrm{i}}={\upbeta}_0+{\upbeta}_{\mathrm{a}}{\mathrm{a}\mathrm{ge}}_{\mathrm{i}}+{\upbeta}_{\mathrm{s}}{\mathrm{s}\mathrm{ex}}_{\mathrm{i}}+{\upgamma}_{\mathrm{c},\mathrm{t},\mathrm{i}}, $$

where *β*_0_, *β*_a_, and *β*_s_ are the ordinary least squares coefficients and the explained variance is estimated as squared Pearson correlation between ranked MSP abundance and residuals *γ*_c,t,i_. Note that this estimate is conservative in the context of a single isolated MSP, as the effect of age and sex is explained away using their full least squared coefficients.

#### Metabolomics statistical analysis

Metabolite intensity was standardized (*z*-scored) across samples. Clusters were obtained using *k*-means, with the number of clusters determined by optimal matching to the molecular classes (from HMDB database) using adjusted random index (ARI) measure. The two dimensional projection was obtained using t-SNE.

Canonical correlation analysis (CCA) between standardized metabolite intensity matrix **M** (321 samples times 1607 metabolite features) and MSP abundance matrix **S** (321 samples times 345 MSPs) was performed by singular value decomposition of the covariance matrix

$$ {\mathbf{UDV}}^{\mathbf{T}}={\mathbf{M}}^{\mathrm{T}}\mathbf{S}, $$

where **U** and **V** are orthonormal matrices of canonical vectors and **D** is the diagonal matrix of explained variances. The significance of canonical vectors was assessed by comparing diagonal entries in **D** against the null distribution, simulated by 1000 random permutations of **M** and **S** and subject to threshold *P* < 0.05. The top 25 canonical vectors in **U**, **V** were then projected to two dimensions using t-SNE.

Metabolite intensities were compared to relative species abundance and cytokine expressions using Spearman correlation subject to *P*_Bonferroni_ < 0.05.

To compare intensity of multiple metabolites with abundance of multiple enzymes, the respective intensities and abundances were averaged and Spearman correlation was calculated.

#### Associations between pathways, genes, and compounds

The KEGG pathways KGML data formats were used to link metabolite and enzyme identifiers with metabolic pathways. The enzyme and pathway identifiers used the KEGG ortholog (ko) format. The metabolite features mapped to 721 KEGG compound identifiers, contained in 350 metabolic pathways. Out of 10,049 enzymes associated with the 350 pathways, 2726 were detected in the stool metagenomes.

## Supplementary Information

**Additional file 1: Supplementary Figures.** This file contains Figures S1-S8.

**Additional file 2: Table S1.** Associations between microbiome clusters 1-3 and subject anthropometric and questionnaire variables. Associations between MSP abundance and subject anthropometric and questionnaire variables (Maaslin 2).

**Additional file 3: Table S2.** Coefficients and significance values for each metagenomic species pangenome (MSP) in the log-linear model of trained and specific immune response.

**Additional file 4: Table S3.** Individual Pearson correlations between MSP abundance ranks and cytokine fold changes, adjusted for age and sex, 2 weeks and 3 months after BCG vaccination.

**Additional file 5: Table S4.** Significant correlations between MSP abundances and metabolite intensities (Spearman correlation, P_Bonferroni_ < 0.05).

**Additional file 6: Table S5.** Phenylalanine metabolism pathway enzyme identity and prevalence detected in the metagenomic assembly encoded by *Bifidobacterium*, *Roseburia*, *Ruminococcus*, *Eubacterium* and *Coprococcus*.

**Additional file 7: Table S6.** List of key resources.

**Additional file 8.** Review history.

## Data Availability

All the datasets and code generated during this study are available at https://gitlab.com/xavier-lab-computation/public/bcg300 [69] and 10.5281/zenodo.5148664 [70], released under the MIT license. The raw metagenomic sequencing reads are available in the NCBI BioProject under identifier PRJNA685797 (https://www.ncbi.nlm.nih.gov/bioproject/PRJNA685797) [71]. This study did not generate new unique reagents; key resources are listed in Additional file 7: Table S6. Further information and requests for resources and reagents should be directed to and will be fulfilled by the corresponding authors.
