# Supplementary material for: The influence of the gut microbiome on BCG-induced trained immunity
Source: Genome Biol. 2021 Sep 22;22:275. doi: 10.1186/s13059-021-02482-0 (PMC8456614; doi:10.1186/s13059-021-02482-0)
Supplement: Supplementary file 1 — Additional file 1: Supplementary Figures. This file contains Figures S1-S8. [file 13059_2021_2482_MOESM1_ESM.pdf]

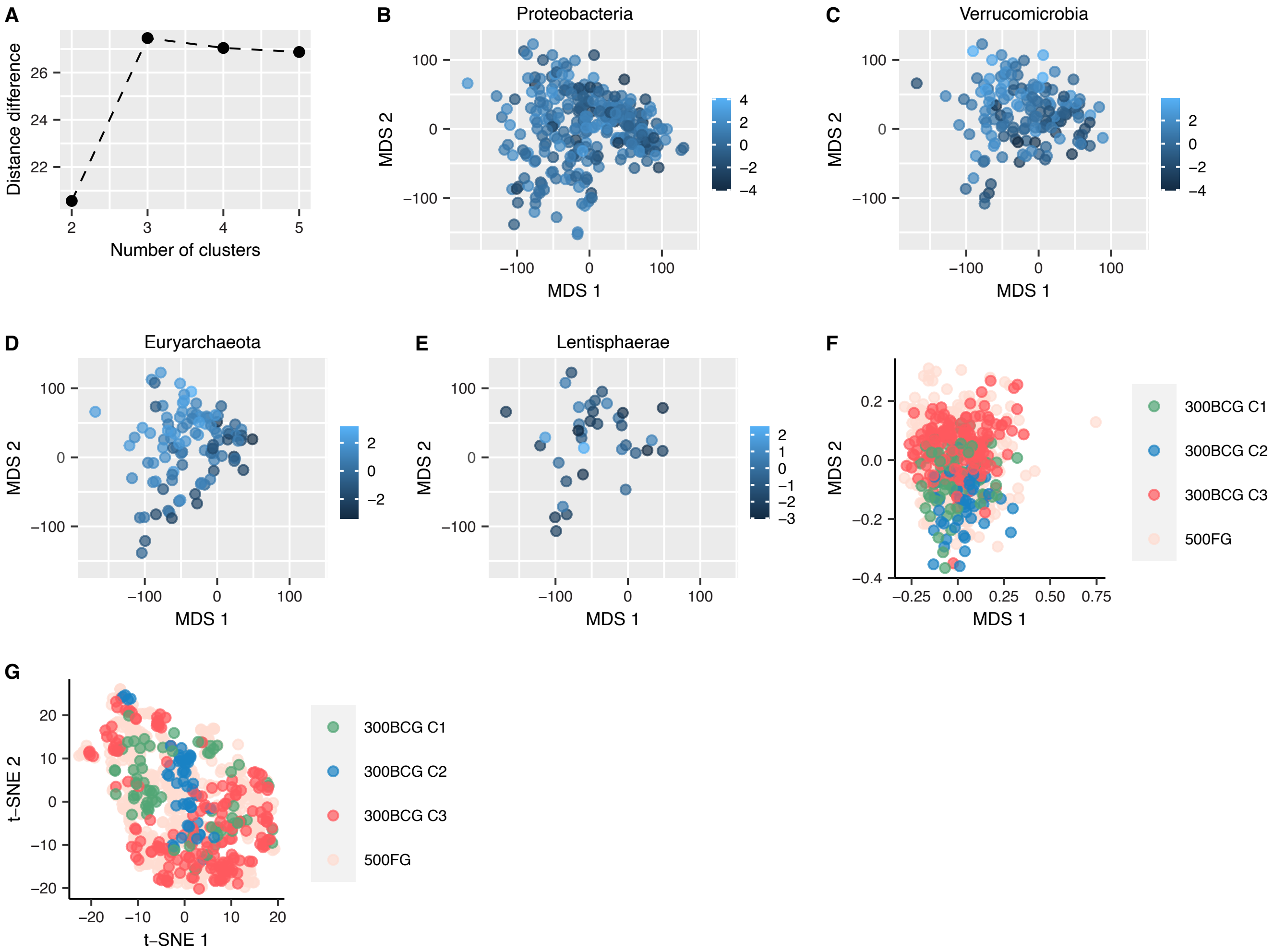

Figure S1. Detailed analysis of metagenomic species clustering. A) Difference between mean between-cluster and within-cluster distance for different numbers of clusters. **B-E)** Abundance of all detected phyla in the cohort. Points where a phylum was not detected were removed from the plots. **F-G)** Comparison with 500FG cohort. MDS and t-SNE plots of Jensen–Shannon distance were based on MetaPhlAn2 species-level relative abundances.

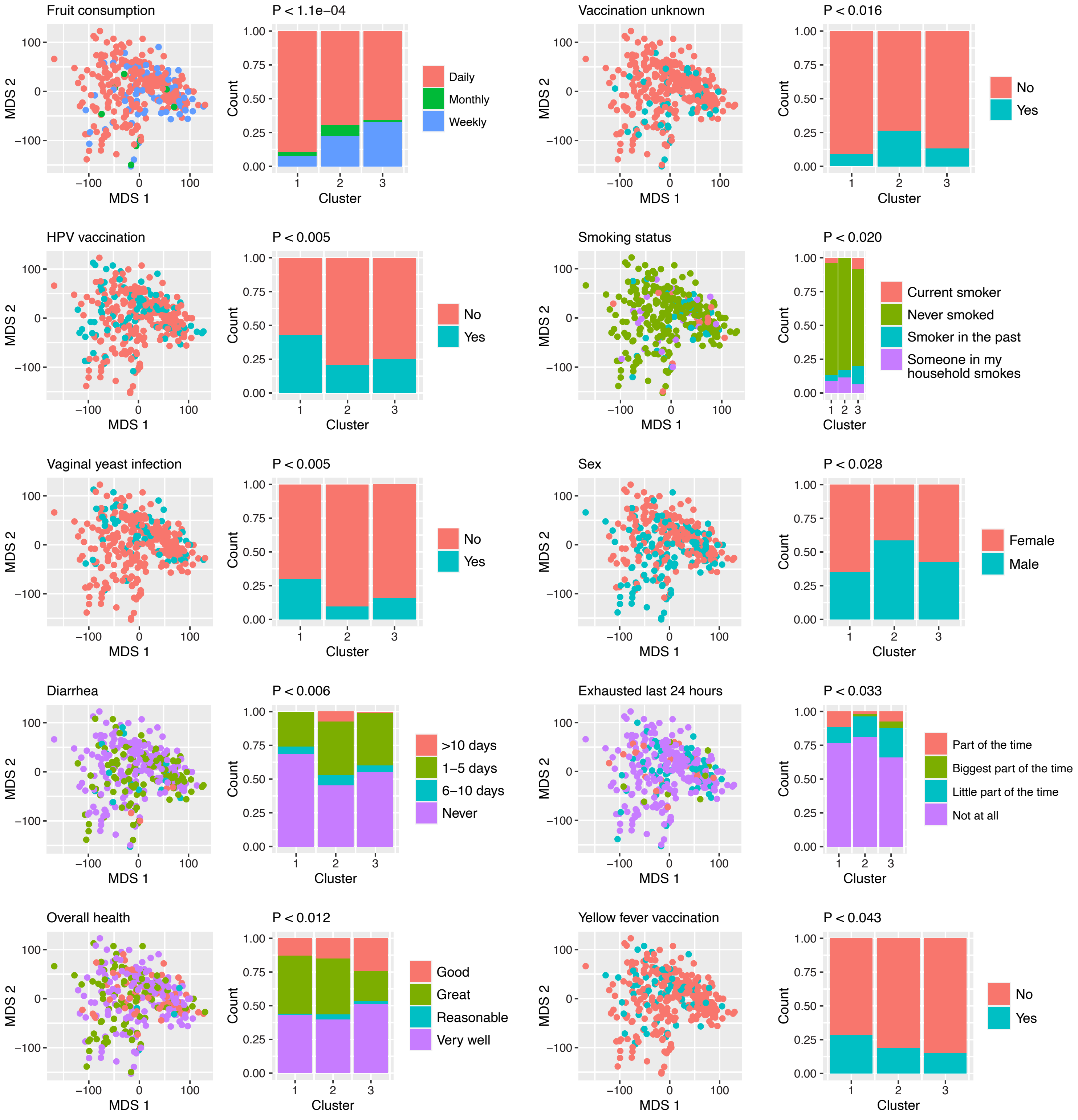

Figure S2. Top ten questionnaire variables associated with three microbiome clusters. Shown are P-values after the Cauchy test.

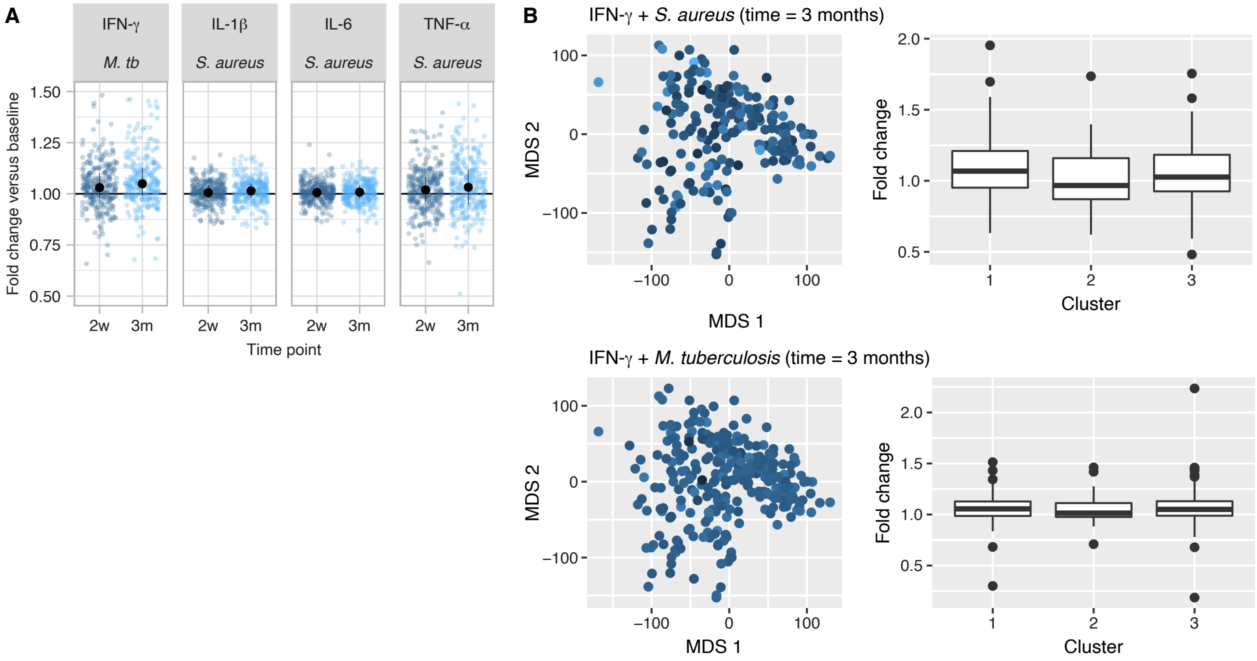

Figure S3. Increase in trained immunity and specific response upon BCG vaccination. A) Fold changes in cytokine expression after 2 weeks and 3 months versus baseline upon *M. tuberculosis* (*M. tb*) or *S. aureus* stimulation. **B)** Assignment of participants into clusters based on core MSP abundances was compared to fold-changes in IFN-γ expression upon *S. aureus* or *M. tuberculosis* stimulation 3 months after BCG vaccination.

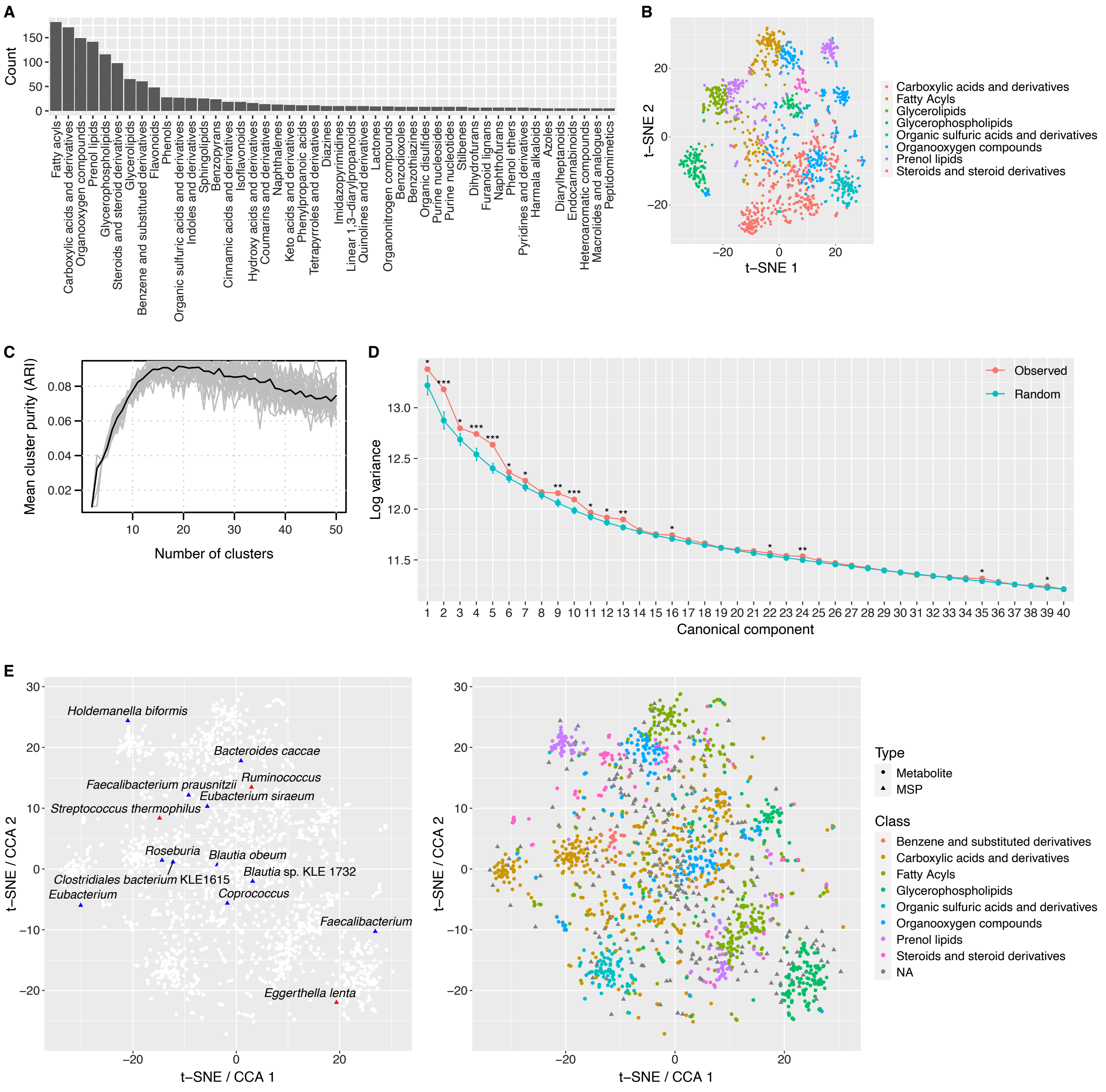

Figure S4. Clustering and quality control of serum metabolomic data. A total of 1,607 m/z peaks were identified by MS/MS and matched to at least one molecule in the Human Metabolome Database (HMDB). In case of multiple matches, the molecule with the least difference in reference and observed m/z was chosen. **A)** The distribution of molecular classes for measured molecules. **B)** Co-abundance clustering. Metabolite intensities were standardized to z-scores across subjects, and clusters were found by the K-means algorithm. As an m/z peak corresponds to multiple candidate molecules, we associated each cluster to the most prevalent molecular class in the cluster. **C)** Number of clusters (20) was selected such that the clusters show maximal correspondence with the molecular classes as measured by the Adjusted Random Index (ARI). The clustering was repeated 50 times for each number of clusters of K with different random initializations. **D)** Canonical correlation analysis (CCA) between 345 microbial species and 1,607 metabolites. Asterisks show significant increase in explained variance of each component compared to null distribution on randomly shuffled data (1,000 shuffles, *P < 0.1, **P < 0.01, ***P < 0.001). **E)** Top 25 canonical components projected with t-SNE, with significant MSPs highlighted, and metabolite classes.

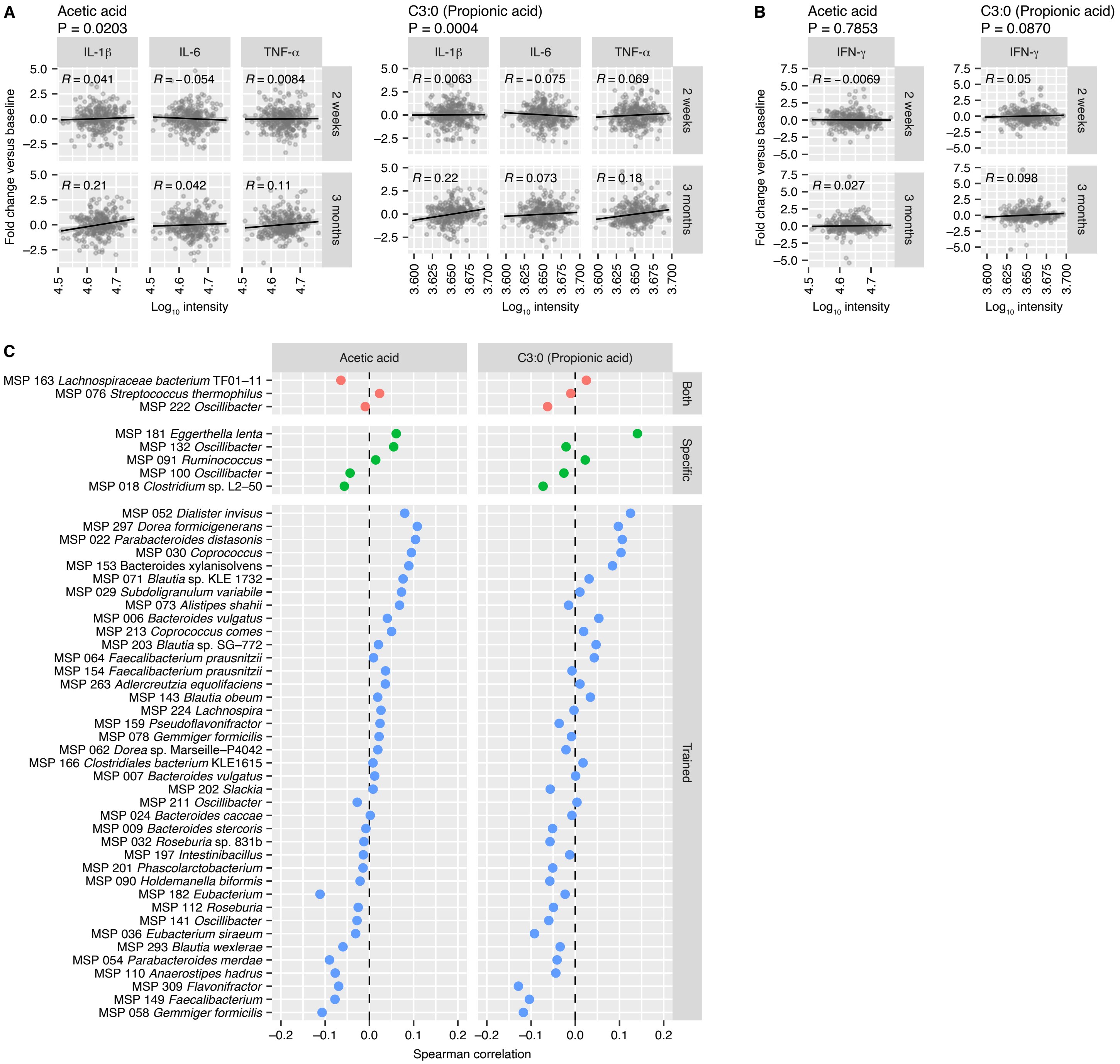

Figure S5. Associations between short-chain fatty acids (SCFAs) acetate and propionate with cytokine responses and MSP abundances. A-B) Associations with acetate and propionate with A) trained immunity and B) specific response. The log-linear model was applied with log-scaled metabolite intensities as covariates and multiple cytokine expressions as response variables. P-values represent significance of association per metabolite. **C)** Spearman correlation coefficients between immunomodulatory MSPs and SCFAs.

A

MSP	Prodigal gene ID	KEGG ko	eggNOG annotation	Seed ortholog e-value
msp_030.core / *Coprococcus*	050_V1_k141_75741_4	K00929	Branched-chain carboxylic acid kinase	2.10e–136
msp_042.core / *Coprococcus eutactus*	148_V1_k141_111583_1	K00929	Branched-chain carboxylic acid kinase	6.70e–175
msp_219.core / *Coprococcus*	179_V1_k141_175108_11	K00929	Branched-chain carboxylic acid kinase	1.40e–177
msp_213.core / *Coprococcus comes*	206_V1_k141_19603_2	K00929	Branched-chain carboxylic acid kinase	1.40e–132
msp_013.core / *Coprococcus eutactus*	215_V1_k141_100015_3	K00929	Branched-chain carboxylic acid kinase	1.40e–175
msp_209.core / *Roseburia*	327_V1_k141_118473_5	K00929	Branched-chain carboxylic acid kinase	2.50e–153

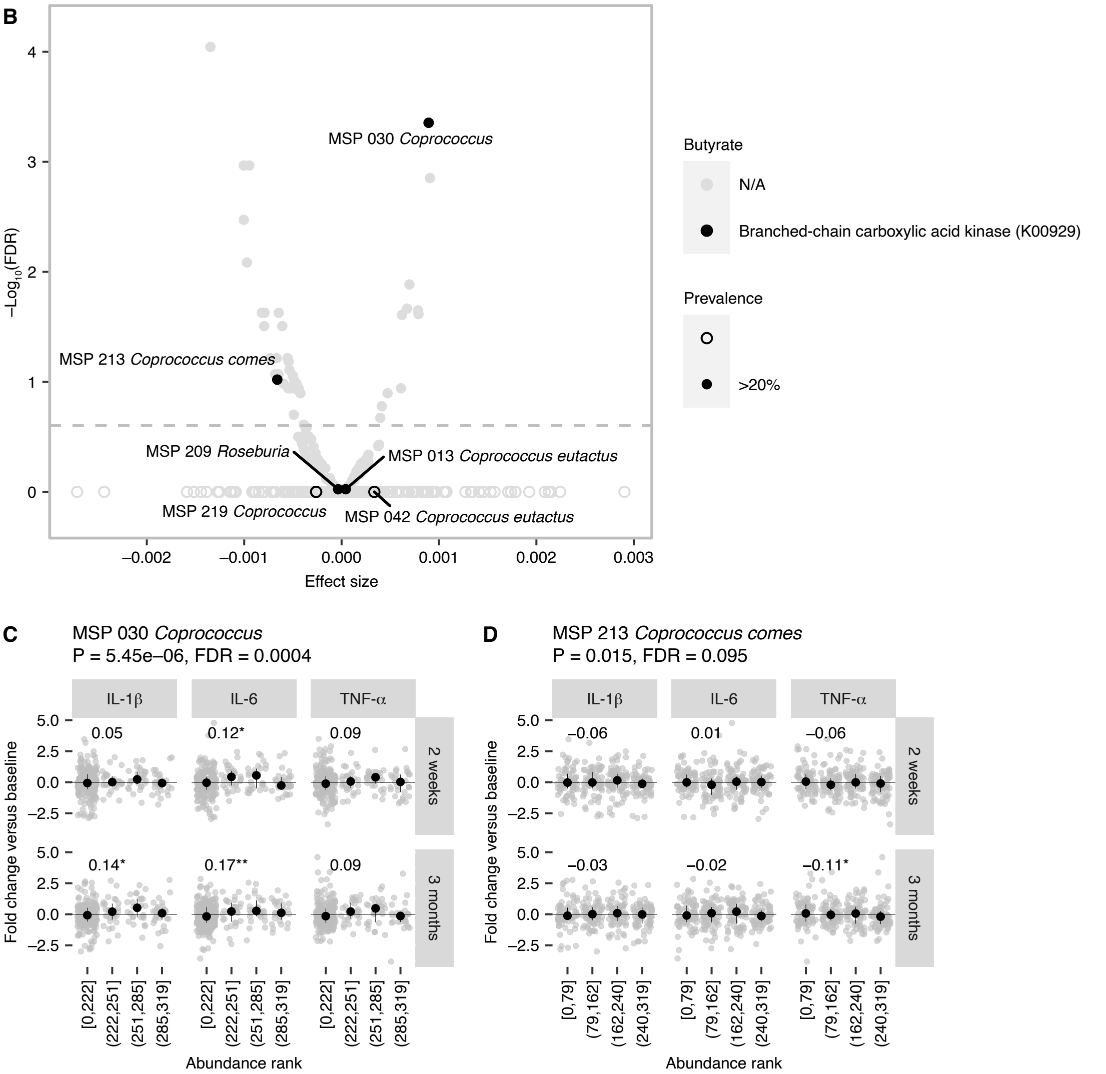

Figure S6. Encoders of butyrate kinase enzyme. A) Gene predictions by Prodigal annotated to the branched-chain carboxylic acid kinase (K00929), the last step in microbial butyrate production. **B)** Significant MSPs associated with trained immune responses subject to FDR < 0.2 and prevalence >20%, adjusted for age and sex (Methods). MSPs encoding a butyrate kinase gene are marked in black. **C)** MSP 030 (*Coprococcus*) and **D)** MSP 213 (*Coprococcus comes*) abundance versus cytokine expression upon *S. aureus* stimulation in the two subsequent time points after vaccination. Relative abundance of the MSP was converted to ranks, and ranks were sorted into bins by equal-frequency binning.

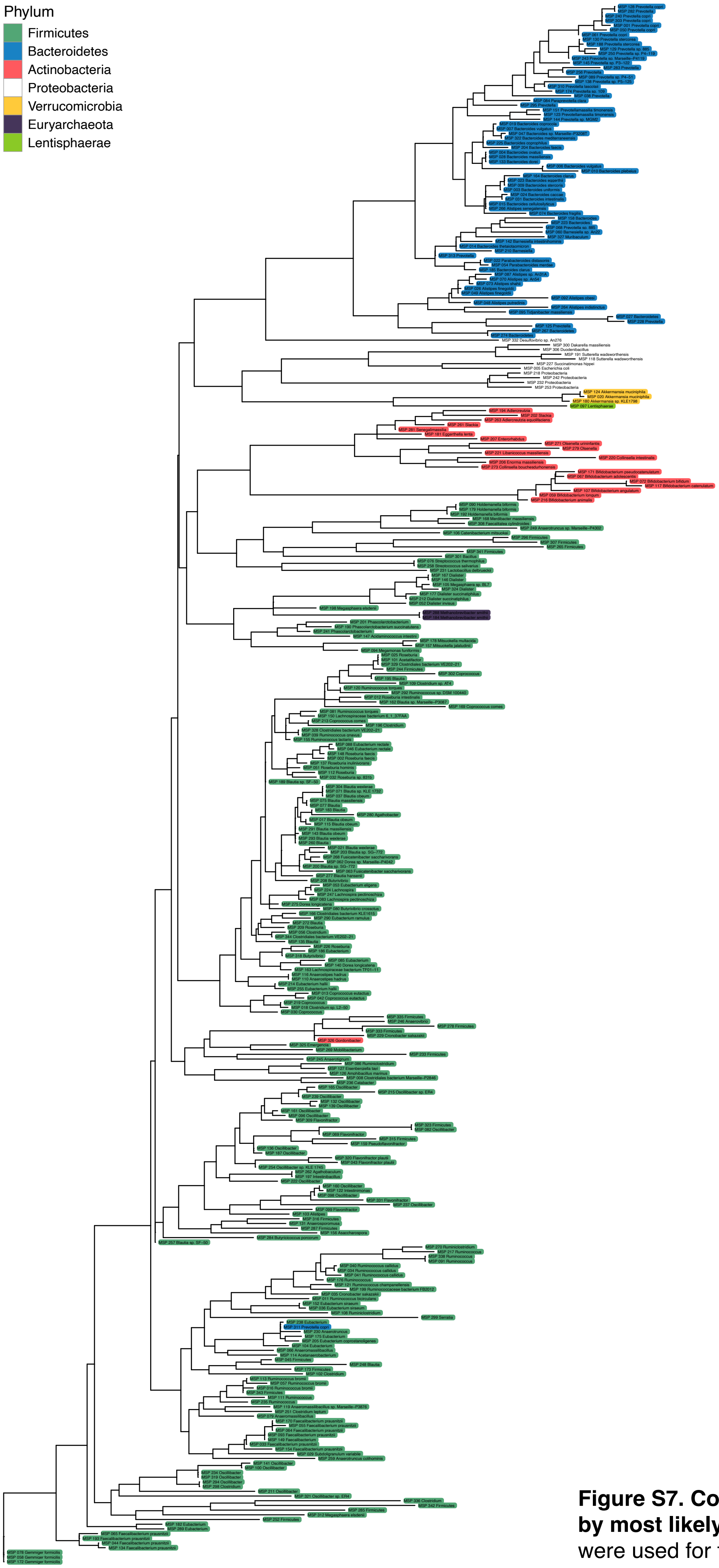

Figure S7. Complete phylogenetic tree of MSPs stratified by most likely phylum-level annotation. Only core genes were used for the alignment with the PhyloPhlAn 3 tool.

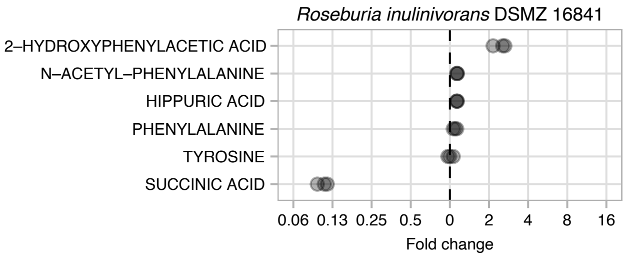

Figure S8. Fold changes relative to media blank controls in measured phenylalanine metabolism compounds intensity for *Roseburia inulinivorans* DSMZ 16841 as retrieved from the Metabolomics Data Explorer (Han et al. 2021).
