# Supplementary material for: The influence of the gut microbiome on BCG-induced trained immunity
Source: Genome Biol. 2021 Sep 22;22:275. doi: 10.1186/s13059-021-02482-0 (PMC8456614; doi:10.1186/s13059-021-02482-0)
Supplement: Supplementary file 8 — Additional file 8. Review history. [file 13059_2021_2482_MOESM8_ESM.docx]

Review History

**First round of review**

**Reviewer 1**

**Are you able to assess all statistics in the manuscript, including the appropriateness of statistical tests used?** Yes, and I have assessed the statistics in my report.

**Comments to author:**

Strazar et al present an interesting and generally well-written and presented manuscript investigating the potential influence of the gut microbiota on BCG induced trained immunity.
Almost nothing is known to date regarding the effects of the microbiota on trained immunity and therefore the study is very timely and novel. There is ever-increasing interest in both trained immunity and the influence of the microbiota on vaccine responses so I expect the study to be of significant interest.
Strengths of the study include investigation of this phenomenon in a very good-sized human cohort (321 adults), though as BCG is usually administered to infants a study in infants would have been even more interesting.
Other strengths include the use of shotgun metagenomics to profile not only the composition of the fecal microbiota but also its encoded functional capacity. The use of untargeted plasma metabolomics is also of significant interest.
There are also however some important weaknesses to be addressed before the study would in my opinion be suitable for publication in Genome Biology.
Throughout the manuscript there is use of language that could be taken to imply causal relationships E.g. line 135 "species were affected by 25 variables"; Line 155 "the effects of microbes on both immune system modalities"; Line 157 "Trained immunity was affected by 40 significant species"; Line 188 "strongly affected the composition of the" ; Line 206 "Roseburia additionally affected three metabolites" etc.
In each case what has been identified is a statistical association/correlation. Please rephrase throughout to avoid statements that imply causality.
In many cases unusually generous FDR thresholds are used in analyses.
E.g. on line 135-136 the authors report that the (relative) abundance of individual taxa were associated with 25 different variables (FDR < 0.25). At a more reasonable threshold of FDR < 0.1 only 1 variable (fruit consummation) is associated with the clusters.
Line 158: FDR < 0.2 used = 40 MSP associated; at FDR < 0.1 = 27 MSPs.
Fig. 2A please clarify log cytokine expression on the y axis. Is this in units; fold-change relative to unstimulated? It would be much clearer to show fold-change in expression at 2w and 3m relative to baseline. It is currently very difficult to assess what the difference is following BCG vaccination.
Given previous reports of BCG inducing trained immunity for 1 year isn't it surprising that there were no differences observed in this study at 3m? Can the authors offer any potential explanations?
Fig. S3 needs a more detailed figure legend including an explanation of what the fold change is in comparison to.
Fig. 2H. It does not appear that the p-values for correlations reported in this figure are corrected for multiple testing and they are extremely weak. After correction I doubt they would be statistically significant.
Line 178: The associations between MSPs in the microbiota and plasma metabolites in no way "validates" or identifies "causal mechanisms". This analysis is very interesting but the authors are overstating what it is capable of showing i.e. that certain taxa are associated both with cytokine expression and with certain plasma metabolites.
Fig. 4D the individual data points are very hard to see. Some of the statistics are also surprising. E.g. the difference in the hisC gene between Bif and Roseburia is highly significant but what looks to be a much bigger difference for the aspB gene is not?
The authors find some interesting links between Roseburia and phenylalanine metabolism. It would significantly strengthen the manuscript if the proposed relationship between Roseburia, phenylalanine and trained immunity was validated in vivo e.g. in a mouse model. For example, the authors could colonise germ free mice with Roseburia (this particular MSP) and assess effects on phenylalanine metabolism and on BCG induced trained immunity.

Minor comments:
Line 89-90 "we identify potential immunomodulatory species that are further validated and filtered by paired plasma metabolomic analysis." This sentence is not very clear especially the use of the words validated and filtered. The metabolomics data does not validate that certain species are immunomodulatory.
Line 723 "Firmicutes contributed to 72.6% of total abundance and prevailed in cluster 1" ◊ Relative abundance has been assessed not abundance. Suggest rephrase The relative abundance of Firmicutes in cluster 1 was 72.6%.
Line 125 "Cluster 1 also showed increased presence of less abundant phyla" ◊ cluster 1 also had an increased relative abundance of phyla…. Technically the abundance of these phyla could be the same across clusters. With the data you have you can only comment on relative abundance.
Line 135 "The shifts in abundance of individual species" ◊ differences in the relative abundance of individual taxa (MSPs?)
Line 160 "More data available on trained immunity …. resulted in greater power". I don't follow the logic of this argument. It doesn't follow that because the authors measured more cytokines for trained vs specific immunity that there is more statistical power for trained immunity…
Line 178 "funnel potential causal mechanisms" - suggest reword "funnel" - identify perhaps.

**Reviewer 2**

**Are you able to assess all statistics in the manuscript, including the appropriateness of statistical tests used?** No, I do not feel adequately qualified to assess the statistics.

**Comments to author:**

Manuscript : « The influence of the gut microbiome on BCG-induced trained immunity »
Strazar et al.

The authors aim at studying the role of the gut microbiome in BCG-induced immune responses including the memory adaptive immune response and the trained immunity. The authors collected stools and blood from a cohort of 321 healthy adult after BCG vaccination and performed metagenomic and metabolomic analyses.
The authors identified microbial species associated with memory adaptive immune response (specific to BCG vaccination) and with trained immune responses (not BCG-specific). In this set-up, 43 immunomodulatory bacteria were identified amongst them 2 were confirmed by both metagenomic and metabolomic analyses. A Roseburia strain negatively associated with trained immunity (BCG-unspecific) and Eggerthella lenta positively associated with memory adaptive immune response. Interesting as these two bacteria have been associated with multiple diseases. Phenylalaninie metabolism was also associated with trained immunity modulation.
The manuscript approaches an emerging research topic focused on the newly described concept of trained immunity. The manuscript brings very interesting and crucial data and shows that microbiota composition might have a role in the development of both specific and trained immune responses induced by BCG Vaccination. The study is well designed, addressed and justified. The methodology is adequate, and results support the main conclusions.

A lot of work and analyses have already been performed in the study. However, some specific points should be clarified to improve the final outcome.

- Trained immunity involved the epigenetic reprogramming of myeloid lineage in the bone marrow. Short chain fatty acids, bacterial metabolites affecting myeloids cells directly impact the acetylation of the histones and have been shown to affect trained immunity in monocytes of obese patients. SCFA-producing bacteria are amongst the species affecting trained and stimulus immune responses. I think it would have been relevant to assess SCFA concentrations in order to correlate these metabolites to metagenomic and cytokine concentration data.

- Hippurate is a metabolite identified in the correlation analyses performed in this paper. This metabolite has been identified as a mediator of metabolic health. Moreover, metabolic syndrome is associated with an increased inflammatory profile. Did the authors have access to the BMI of the donors and correlate the BMI to cytokines, metagenomic and metabolomic profiles ?

- PRRs and consequently MAMPs are a trigger of trained immunity. Commensal bacteria composition impacts the intestinal permeability and consequently circulating MAMPs concentration such as LPS. Circulating LPS and LBP can be monitored. Can the authors discuss this point ?

**Response to Reviewers**

**Reviewer reports: Reviewer #1**

Strazar et al present an interesting and generally well-written and presented manuscript investigating the potential influence of the gut microbiota on BCG induced trained immunity. Almost nothing is known to date regarding the effects of the microbiota on trained immunity and therefore the study is very timely and novel. There is ever-increasing interest in both trained immunity and the influence of the microbiota on vaccine responses so I expect the study to be of significant interest. Strengths of the study include investigation of this phenomenon in a very good-sized human cohort (321 adults), though as BCG is usually administered to infants a study in infants would have been even more interesting. Other strengths include the use of shotgun metagenomics to profile not only the composition of the fecal microbiota but also its encoded functional capacity. The use of untargeted plasma metabolomics is also of significant interest. There are also however some important weaknesses to be addressed before the study would in my opinion be suitable for publication in Genome Biology.

Remark 1. Throughout the manuscript there is use of language that could be taken to imply causal relationships E.g. line 135 "species were affected by 25 variables"; Line 155 "the effects of microbes on both immune system modalities"; Line 157 "Trained immunity was affected by 40 significant species"; Line 188 "strongly affected the composition of the" ; Line 206 "Roseburia additionally affected three metabolites" etc. In each case what has been identified is a statistical association/correlation. Please rephrase throughout to avoid statements that imply causality.

**Answer: We have modified these and similar statements to state association or correlation.**

Remark 2. In many cases unusually generous FDR thresholds are used in analyses. E.g. on line 135-136 the authors report that the (relative) abundance of individual taxa were associated with 25 different variables (FDR < 0.25). At a more reasonable threshold of FDR < 0.1 only 1 variable (fruit consummation) is associated with the clusters. Line 158: FDR < 0.2 used = 40 MSP associated; at FDR < 0.1 = 27 MSPs.

**Answer: We agree with the Reviewer on limitations of the associations detected by the applied methods and thresholds. We opted to report subject-related variables based on an unadjusted test in order to convey more information, as we do not analyze these associations further.**

**The microbiome correlations are corrected for multiple testing based on permissive values used in the metagenomics analysis. The selection is related to the challenges in correlating loosely connected environments: ex vivo stimulated cells and gut microbes, as noted previously (1). Similar values were used in previous studies correlating microbes and blood markers (2, 3).**

Remark 3. Fig. 2A please clarify log cytokine expression on the y axis. Is this in units; fold-change relative to unstimulated? It would be much clearer to show fold-change in expression at 2w and 3m relative to baseline. It is currently very difficult to assess what the difference is following BCG vaccination.

**Answer: The cytokine concentrations were measured in pg / mL, adjusted for plate variation and log-transformed. Therefore, the units are log-transformed residuals of concentrations adjusted for plate variation using a linear model. We have included these details to the Methods section. The fold changes between two subsequent time points and the baseline were included in Fig. S3A.**

Remark 4. Given previous reports of BCG inducing trained immunity for 1 year isn't it surprising that there were no differences observed in this study at 3m? Can the authors offer any potential explanations?

**Answer: We thank the Reviewer for noticing the missing statement in the results. The increase in cytokine response was significant at both two weeks and three months after BCG vaccination. This was assessed by Kruskal-Wallis test; hence a single significance value was reported for each stimulus. We have updated the interpretation of these results and reported the exact P-values on Fig. 2A.**

Remark 5. Fig. S3 needs a more detailed figure legend including an explanation of what the fold change is in comparison to.

**Answer: The figure legend was updated. Assignment of participants into clusters based on core MSP abundances was compared to fold changes in IFN-γ expression upon S. aureus and M. tuberculosis stimulation after 2 weeks and 3 months from BCG vaccination.**

Remark 6. Fig. 2H. It does not appear that the p-values for correlations reported in this figure are corrected for multiple testing and they are extremely weak. After correction I doubt they would be statistically significant.

**Answer: We agree with the Reviewer that significance values in Figure 2 warrant clarification. The selection of immunomodulatory MSPs was performed by using trained immunity and specific response linear models (Fig. 2D-E), where the selection was corrected for multiple testing using FDR across all prevalent microbes. The model tests for significance of a change in all cytokines simultaneously.**

**The relevant significance values were added to Fig. 2H-I. Each individual panel also shows unadjusted Spearman correlation coefficients to further quantify the change of each individual cytokine and time point.**

Remark 7. Line 178: The associations between MSPs in the microbiota and plasma metabolites in no way "validates" or identifies "causal mechanisms". This analysis is very interesting but the authors are overstating what it is capable of showing i.e. that certain taxa are associated both with cytokine expression and with certain plasma metabolites.

**Answer: We agree with the reviewer and have reworded the relevant statement to suggest that the increased associations between immunomodulatory microbes and metabolites corroborates, rather than validates, cytokine associations.**

Remark 8. Fig. 4D the individual data points are very hard to see. Some of the statistics are also surprising. E.g. the difference in the hisC gene between Bif and Roseburia is highly significant but what looks to be a much bigger difference for the aspB gene is not?

**Answer: The figure was updated according to the suggestions by the Reviewer to more clearly display the individual data points. We compared the abundance to all four of the genera and displayed the explicit p-value with the closest genus for genes where Roseburia encode with the highest abundance: hisC, mhpE, yhDR, paaH, paaF, paaI, and enr. The figure caption states that all the comparisons are one sided, i.e. testing for increased abundance in Roseburia.**

Remark 9. The authors find some interesting links between Roseburia and phenylalanine metabolism. It would significantly strengthen the manuscript if the proposed relationship between Roseburia, phenylalanine and trained immunity was validated in vivo e.g. in a mouse model. For example, the authors could colonise germ-free mice with Roseburia (this particular MSP) and assess effects on phenylalanine metabolism and on BCG induced trained immunity.

**Answer: We agree with the Reviewer that the observed links between Roseburia, phenylalanine metabolism and trained immunity warrant further investigation. In order to complete the revision in due time, we outlined additional experimental evidence for the role of Roseburia in phenylalanine metabolism. We queried recent data from in vitro culture experiments with targeted, standard-based metabolomics of microbial supernatants (4). The only Roseburia representative was R. inulinivorans, which is a close relative to Roseburia MSP 112 and caused at least two-fold change as follows:**

**● An increase in 2-hydroxyphenylacetate, which is downstream of the yhDR and hisC genes.**

**● A decrease in succinate, which is upstream of the mpH gene.**

**The direction of changes is aligned with the enzymatic functions in the phenylalanine metabolism pathway and supports an active role of Roseburia in phenylalanine metabolism pathways.**

**A supernatant from a related Roseburia (R. intestinalis), shown to contain butyrate and propionate, can cause a decrease of IL-6 upon LPS stimulation (5). We observed no correlation between butyrate or propionate with Roseburia genomes detected here (see Reviewer 2, Remark 1); however, this does not exclude the possibility of an immunomodulatory mechanism independent of SCFAs.**

**We have added Figure S8 to show fold changes in measured metabolite intensity for compounds in the phenylalanine metabolism pathway and text describing our interpretation of the results.**

Minor remarks:

Line 89-90 "we identify potential immunomodulatory species that are further validated and filtered by paired plasma metabolomic analysis." This sentence is not very clear, especially the use of the words validated and filtered. The metabolomics data does not validate that certain species are immunomodulatory.

**Answer: We reworded the sentence to suggest support rather than validation of the observations.**

Line 723 "Firmicutes contributed to 72.6% of total abundance and prevailed in cluster 1" Relative abundance has been assessed not abundance. Suggest rephrase The relative abundance of Firmicutes in cluster 1 was 72.6%.

**Answer: The sentence was reworded as recommended.**

Line 125 "Cluster 1 also showed increased presence of less abundant phyla" cluster 1 also had an increased relative abundance of phyla…. Technically the abundance of these phyla could be the same across clusters. With the data you have you can only comment on relative abundance.

Line 135 "The shifts in abundance of individual species" differences in the relative abundance of individual taxa (MSPs?)

**Answer: The sentences were reworded to emphasize the relative abundance nature of the data.**

Line 160 "More data available on trained immunity …. resulted in greater power". I don't follow the logic of this argument. It doesn't follow that because the authors measured more cytokines for trained vs specific immunity that there is more statistical power for trained immunity…

**Answer: The interpretation was rewritten to omit the notion of statistical power and emphasize that the trained immunity is measured with more biological markers than specific response, and hence more species are identified under the same thresholds.**

Line 178 "funnel potential causal mechanisms" - suggest reword "funnel" - identify perhaps.

**Answer: The sentence was rephrased accordingly.**

**Reviewer #2:**

The authors aim at studying the role of the gut microbiome in BCG-induced immune responses including the memory adaptive immune response and the trained immunity. The authors collected stools and blood from a cohort of 321 healthy adult after BCG vaccination and performed metagenomic and metabolomic analyses.

The authors identified microbial species associated with memory adaptive immune response (specific to BCG vaccination) and with trained immune responses (not BCG-specific). In this setup, 43 immunomodulatory bacteria were identified amongst them 2 were confirmed by both metagenomic and metabolomic analyses. A Roseburia strain negatively associated with trained immunity (BCG-unspecific) and Eggerthella lenta positively associated with memory adaptive immune response. Interesting as these two bacteria have been associated with multiple diseases. Phenylalaninie metabolism was also associated with trained immunity modulation.

The manuscript approaches an emerging research topic focused on the newly described concept of trained immunity. The manuscript brings very interesting and crucial data and shows that microbiota composition might have a role in the development of both specific and trained immune responses induced by BCG Vaccination. The study is well designed, addressed and justified. The methodology is adequate, and results support the main conclusions. A lot of work and analyses have already been performed in the study. However, some specific points should be clarified to improve the final outcome.

Remark 1. Trained immunity involved the epigenetic reprogramming of myeloid lineage in the bone marrow. Short chain fatty acids, bacterial metabolites affecting myeloids cells directly impact the acetylation of the histones and have been shown to affect trained immunity in monocytes of obese patients. SCFA-producing bacteria are amongst the species affecting trained and stimulus immune responses. I think it would have been relevant to assess SCFA concentrations in order to correlate these metabolites to metagenomic and cytokine concentration data.

**Answer: We have detected metabolomic features in untargeted metabolomics related to following SCFAs:**

**C2H4O2 -H(-) 100 Acetic acid**

**C3H6O2 -H(-) 100 C3:0 (Propionic acid)**

**Upon applying the log linear model of trained immunity, both were positively associated with the trained immunity response: acetate (P = 0.02) and propionate (P=0.0004). Of the three well-known SCFAs acetate, propionate and butyrate, only the latter is known to affect myeloid lineage differentiation through histone deacetylation (6).**

**While butyrate was not detected in plasma samples with metabolomic methods at our disposal, we looked for encoders of butyrate kinase in the assembled metagenomic species. The enzyme was found in six species, including Roseburia MSP 209 and two Coprococcus species, MSP 030 and MSP 213, associated with increased and decreased trained immunity response.**

**We have added these results as supplementary material. Figure S5 now shows associations between cytokine responses, immunomodulatory microbes and SCFAs (acetate and propionate). Figure S6 includes the effects of butyrate kinase encoders on trained immunity.**

Remark 2. Hippurate is a metabolite identified in the correlation analyses performed in this paper. This metabolite has been identified as a mediator of metabolic health. Moreover, metabolic syndrome is associated with an increased inflammatory profile.

**Answer: The body mass index (BMI) was assessed for all the participants. The cohort is largely composed of healthy young adults (median age 23), 85% of which have a BMI under 25 (boundary between ‘healthy’ and ‘overweight’). Given the very homogeneous distribution, it is unsurprising we found no associations between BMI and cytokine responses or microbial abundances. We have added the relevant remark to the Discussion.**

Remark 3. PRRs and consequently MAMPs are a trigger of trained immunity. Commensal bacteria composition impacts the intestinal permeability and consequently circulating MAMPs concentration such as LPS. Circulating LPS and LBP can be monitored. Can the authors discuss this point ?

**Answer: We thank the Reviewer for pointing out the relevant pathway in inducing myeloid cell response through engagement of pattern recognition receptors and bacterial products or cell wall components, such as LPS or muramyl dipeptide (MDP). The Discussion was extended to include references to LPS-induced immune tolerance and data on MDPinduced trained immunity in humans, which point to exposure to MDP being positively associated with immune response.**

**References**

**1. Schirmer M, Smeekens SP, Vlamakis H, Jaeger M, Oosting M, Franzosa EA, et al. Linking the Human Gut Microbiome to Inflammatory Cytokine Production Capacity. Cell. 2016;167(4):1125-36 e8.**

**2. Lloyd-Price J, Arze C, Ananthakrishnan AN, Schirmer M, Avila-Pacheco J, Poon TW, et al. Multi-omics of the gut microbial ecosystem in inflammatory bowel diseases. Nature. 2019;569(7758):655-62.**

**3. Asnicar F, Berry SE, Valdes AM, Nguyen LH, Piccinno G, Drew DA, et al. Microbiome connections with host metabolism and habitual diet from 1,098 deeply phenotyped individuals. Nat Med. 2021;27(2):321-32.**

**4. Han S, Van Treuren W, Fischer CR, Merrill BD, DeFelice BC, Sanchez JM, et al. A metabolomics pipeline for the mechanistic interrogation of the gut microbiome. Nature. 2021;595(7867):415-20.**

**5. Luo W, Shen Z, Deng M, Li X, Tan B, Xiao M, et al. Roseburia intestinalis supernatant ameliorates colitis induced in mice by regulating the immune response. Mol Med Rep. 2019;20(2):1007-16.**

**6. Chang PV, Hao L, Offermanns S, Medzhitov R. The microbial metabolite butyrate regulates intestinal macrophage function via histone deacetylase inhibition. Proc Natl Acad Sci U S A. 2014;111(6):2247-52.**

**Second round of review**

**Reviewer 1**

The authors have satisfactorily addressed my comments. I congratulate them on a nice paper and look forward to seeing it published.
